# From Design to Single-Cell Cloning: A Complete RNP-Based CRISPR-Cas9 Protocol for Precision Gene Correction in Human iPSCs

**DOI:** 10.1007/s12015-026-11221-6

**Published:** 2026-08-31

**Authors:** Giacomo Roman, Knut H. Lauritzen, Barbora Smolkova, Runar Almaas, Per Morten Sandset, Benedicte Stavik, Gareth J. Sullivan, Maria E. Chollet

**Affiliations:** 1 Institute of Clinical Medicine, University of Oslo, Oslo, Norway; 2 Department of Haematology, Oslo University Hospital, Oslo, Norway; 3 Research Institute of Internal Medicine, Oslo University Hospital, Oslo, Norway; 4 Department of Pediatric Research, Oslo University Hospital, Oslo, Norway; 5 School of Medicine, University of St Andrews, St Andrews, UK

**Keywords:** CRISPR-Cas9, iPSCs, Gene editing protocol, Electroporation, Single-cell cloning

## Abstract

Precise genome editing of induced pluripotent stem cells (iPSCs) using clustered regularly interspaced short palindromic repeats and CRISPR-associated protein 9 (CRISPR-Cas9) has opened unprecedented avenues for advancements in regenerative medicine and disease modelling. However, the establishment of isogenic single-cell-derived iPSC populations, particularly upon CRISPR-Cas9 gene editing modifications, is one of the major challenges still associated with these advanced methodologies relying on low-rate editing events and requiring defined clonogenicity. In response, we have developed a systematic, comprehensive and efficient workflow combining generation, genotyping and expansion of high-quality monoclonal iPSC lines following CRISPR-Cas9 genome editing. In particular, the protocol incorporates optimized single-cell cloning procedures for two commercially available dispensing platforms, one based on microfluidic imaging and the other on impedance technology, together with rapid droplet digital PCR (ddPCR)-based screening of non-homologous end joining (NHEJ) and homology-directed repair (HDR) outcomes. By combining gentle single-cell handling with advanced genotyping methodologies, the protocol enables efficient early assessment of editing outcomes before commitment to labour-intensive clonal derivation, thereby accelerating project timelines, minimising cell stress and loss, preserving genetic fidelity and supporting scalability. Coupled with precisely defined culture conditions tailored for post-seeding recovery, these approaches aim to improve iPSC viability and clonal outgrowth, achieving at least 60% in 96-well plate format within 10 days. Importantly, the protocol goes beyond step-by-step experimental instructions by providing comprehensive design strategies, decision-making criteria, and practical advice for avoiding and addressing the common pitfalls and unintended consequences of these advanced methods. Collectively, this integrated end-to-end approach provides a robust and high-throughput framework for the reliable production of monoclonal genome-edited iPSCs, thereby advancing translational research and the development of iPSC-based regenerative therapies and disease models.

**Graphical Abstract:**

**Figure:**
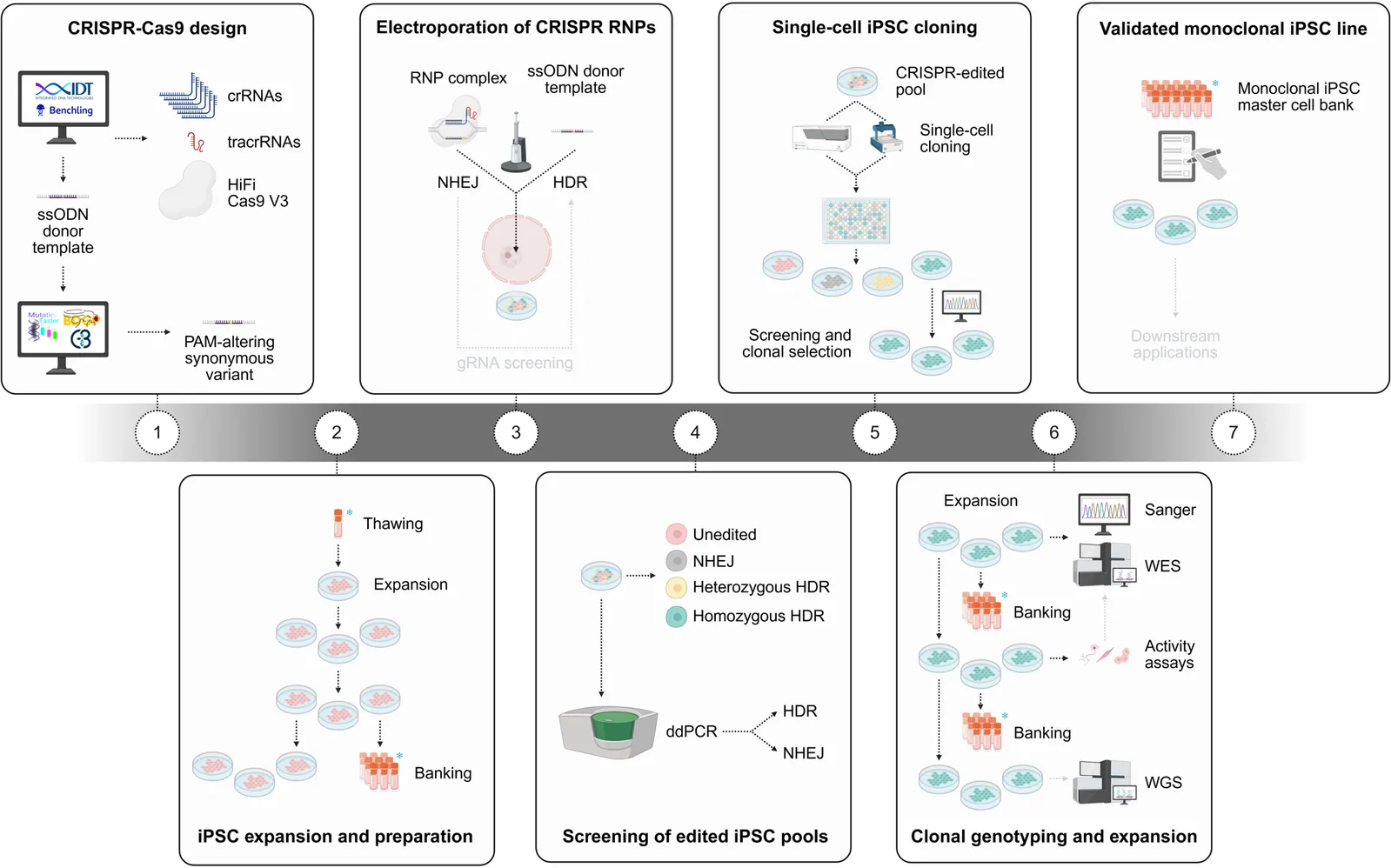

**Supplementary Information:**

The online version contains supplementary material available at https://doi.org/10.1007/s12015-026-11221-6.

## Abstract (--)

This integrated end-to-end protocol provides a comprehensive and efficient workflow for the generation, screening and expansion of high-quality monoclonal iPSC lines after CRISPR-Cas9 editing.

## Introduction

Since its breakthrough in 2006 by Takahashi and Yamanaka [1, 2], induced pluripotent stem cell (iPSC) technology has led to unprecedented progress in regenerative medicine, cell therapy, disease modelling and drug discovery [3, 4]. iPSCs are generated by reprogramming somatic cells into an embryonic stem cell-like pluripotent state through the ectopic expression of four different transcription factors, SRY-box transcription factor 2 (SOX2), octamer-binding transcription factor 4 (OCT4), Krüppel-like factor 4 (KLF4) and Myc proto-oncogene (c-MYC) [4]. The pluripotency and self-renewal capacity of iPSCs allow for the generation of unlimited amounts of virtually any differentiated human cell type, either as two-dimensional cultures or more advanced, complex multicellular three-dimensional organoids [5–7]. In parallel, the advent of clustered regularly interspaced short palindromic repeats (CRISPR)-Cas9 programmable genome editing has transformed the landscape of biomedical research by providing the ability to accurately and precisely modify the human genome and, in particular, to directly introduce or correct specific mutations in iPSCs [8–11]. Together, they allow the generation of isogenic cellular models and facilitate precise gene correction in patient-derived cells, thereby accelerating translational research and the development of personalised, autologous regenerative therapies. Mechanistically, the conventional CRISPR-Cas9 system introduces a site-specific double-strand break (DSB) in the target DNA sequence, three base pairs upstream of the consensus sequence known as the protospacer adjacent motif (PAM) [9, 12, 13]. The resulting DSB is then repaired through either non-homologous end joining (NHEJ) or homology-directed repair (HDR). NHEJ is the predominant, error-prone pathway that rejoins the two DNA ends by introducing small insertions or deletions. HDR, in contrast, enables precise sequence correction or insertion using a homologous donor template, but depends on cell cycle and occurs at very low frequency. To increase the efficiency of precise HDR-mediated repair, various strategies have been explored to bias cellular repair toward HDR, such as optimisation of the synthetic donor template and small guide RNA (sgRNA) chemistry and delivery, transient inhibition of key NHEJ factors and modulation of HDR mediators [14–21].

However, following CRISPR-mediated genome editing, iPSC populations inevitably become genetically heterogeneous due to the difference in editing efficiency in each individual cell [10, 22–25]. To ensure both the genetic integrity and safety of downstream applications, it is therefore essential to isolate and expand single-cell-derived clones that are carrying only the intended modification. Nevertheless, establishing such monoclonal CRISPR-edited iPSC lines, which are a prerequisite for syngeneic and genetically defined populations for both disease modelling and clinical translation, represents a significant technical challenge [24]. Conventional isolation methods, including manual colony picking or fluorescence-activated cell sorting (FACS)-based single-cell isolation, combined with conventional culture techniques, are often inefficient or overly harsh, resulting in reduced clonal viability, poor recovery and suboptimal cloning efficiency [22, 24]. To overcome these limitations, we established a rapid and highly efficient protocol for accurate and precise genome editing and single-cell cloning of iPSC populations. The protocol implements transient delivery of ribonucleoprotein (RNP) complexes that have been shown to have immediate nuclease activity and rapid clearance from the cell, thereby maximising on-target efficiency and minimising off-target risks [26–28]. Furthermore, the workflow incorporates two automated single-cell dispensing technologies, providing high-throughput, accurate, rapid and gentle single-cell isolation. The protocol also includes a stepwise genotyping and validation framework to continuously monitor the genome editing outcome. Droplet digital polymerase chain reaction (ddPCR) is used to rapidly and quantitatively assess both NHEJ and HDR outcomes at the pool level, enabling selection of the most efficiently edited population for subsequent single-cell isolation. Immediately after clonal expansion, Sanger sequencing-based screening is used to confirm on-target correction, while whole-exome sequencing (WES) provides a comprehensive evaluation of genomic integrity and off-target effects. Throughout the process, the real-time analytical pipeline enables early decision-making to ensure that only correctly edited, genomically stable clones are advanced and expanded [25].

## Expected Outcome

We present here a comprehensive protocol that has been successfully applied across multiple iPSC lines, genes and target mutations. In contrast to protocols addressing only one or a few selected steps of the workflow, our approach integrates and optimises multiple advanced methodologies into a unified and practical framework, providing an end-to-end solution spanning editing design, clonal isolation, screening, validation of editing outcomes and clonal expansion. By combining efficient editing strategies with rigorous downstream characterisation, our protocol is designed to improve reliability, reproducibility, scalability and clonal quality across a broad range of genome-editing settings and applications. In addition to detailed experimental instructions, we provide design strategies, recommendations, representative examples and troubleshooting guidance spanning the entire process from experimental planning to single-cell cloning. We also outline how to prevent and address the major pitfalls associated with each step of the workflow, while offering practical approaches for the long-term maintenance of genetically stable, high-quality iPSC clones suitable for countless basic and translational applications.

Although tailored for HDR-based correction strategies, the flexibility of this protocol ensures its applicability across diverse genetic editing contexts, including gene knockout (KO) via either NHEJ or HDR. It thus suits a broad range of applications, including therapeutic gene correction in patient-derived iPSCs and the generation of isogenic disease models. As a practical example detailed in this paper, the protocol describes the correction of a homozygous missense mutation in exon 8 of the *F7* gene, specifically the guanine (G) to cytosine (C) substitution at position 718 (c.718G > C, NM_000131.4; Hg38 chr13: g.113117509G > C), which has been reported in Roman et al., 2026 [29]. Correction of this specific mutation in patient-derived iPSCs demonstrates the efficiency and precision of the workflow, from guide RNA (gRNA) and homology-directed repair (HDR) donor template design through electroporation, single-cell cloning and comprehensive genetic validation.

### Design of Guide RNAs and Donor Template

Timing: 1 day.

### Procedure

**Fig. 1:**
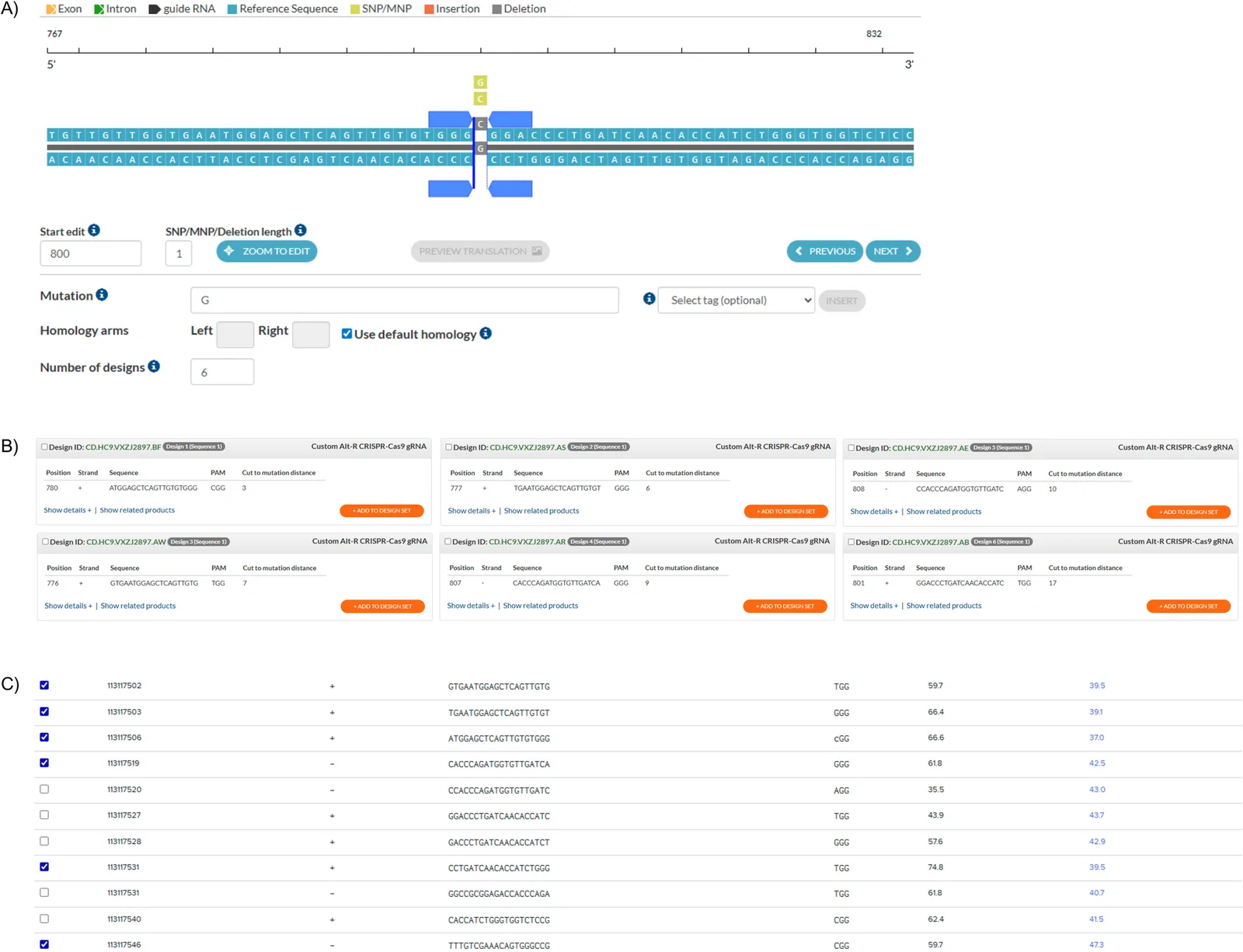
Design of CRISPR gRNAs. (**A**) Design of the therapeutic gene edit c.718 C > G (NM_000131.4) with the Alt-R HDR Design Tool. (**B**) Design of six different gRNAs with the Alt-R HDR Design Tool software. (**C**) Selection of the previously designed CRISPR gRNA in Benchling

1. Use Ensembl genome browser to retrieve the gene summary, chromosomal coordinates, and Ensembl transcript ID.
2. Access Benchling, then navigate to: “Create” > “CRISPR” > “CRISPR guides”. Input the Ensembl gene ID (and confirm that genome assembly, Ensembl gene ID and transcript ID are accurate before proceeding). In addition, make sure to select the correct genome (GRCh38, hg38, homo sapiens) and keep the default CRISPR guide design parameters. Visualise the sequence map (Supplementary Fig. 1 A).
3. If performing a therapeutic CRISPR correction, identify the target nucleotide and introduce the disease-associated mutation using keyboard input (Supplementary Fig. 1B and C).
4. Open the online Alt-R HDR Design Tool software (Integrated DNA Technologies, IDT) and input the desired target sequence using one of the following entry formats: “gene symbol”, “coordinate input/accession number”, or “sequence”. For therapeutic correction of a disease-associated mutation (Fig. 1A), use the “sequence” input format and paste in the target sequence from Benchling, which has been modified in step 3 to include the disease variant.

5. Review the displayed sequence and select the exact nucleotide position to edit. Insert the desired base change (Fig. 1A).

Note: Keep the default IDT homology-arm setting, i.e., homology symmetric arms of 40 bp on the left and 40 bp on the right. When introducing mutations on a wild-type background, IDT allows the possibility to add silent variants, which include both PAM-altering and repair-track mutations.

**Fig. 2:**
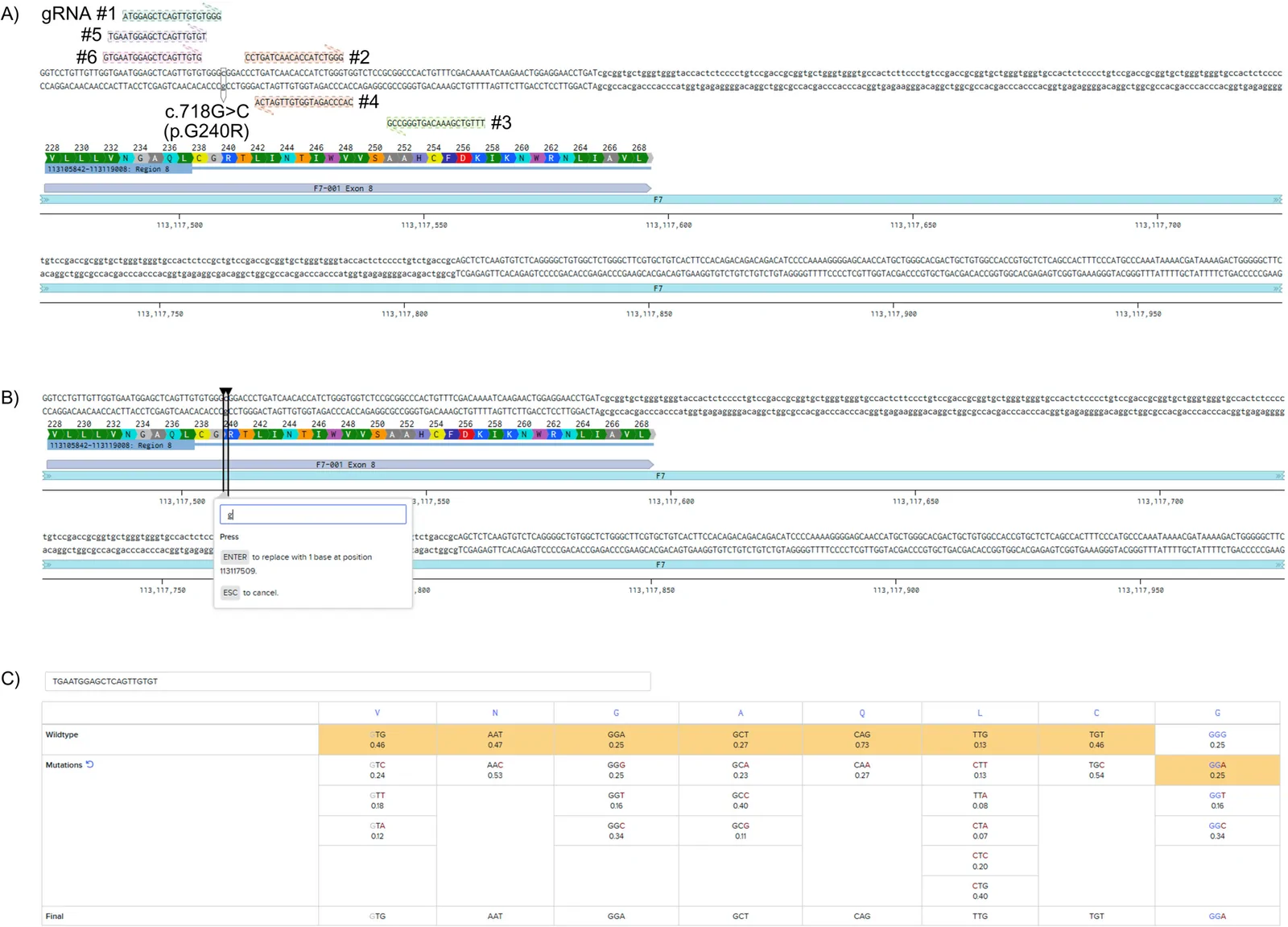
Schematic overview of the editing strategy. (**A**) Overview and visualisation of the selected gRNA on the gene map in Benchling. (**B**) Introduction of the intended gene edit into the mutant sequence for the design of the PAM-altering synonymous variants. (**C**) PAM-altering alternatives for gRNA #5. The PAM-modifying synonymous variant for gRNA #5 was designed as c.717G > A (NM_000131.4; GRCh38 chr13: g.113117508G > A). PAM bases are coloured blue

Note: The designed sequence corresponds to the target-specific CRISPR RNA (crRNA) that is ordered as a synthetic RNA oligonucleotide and subsequently annealed to the universal Alt-R CRISPR-Cas9 tracrRNA (67-mer) to form the functional Alt-R CRISPR-Cas9 crRNA: tracrRNA duplex for RNP assembly.

Note: ssODN donors complementary to the targeting strand are recommended for PAM-proximal edits, and to the non-targeting strand for PAM-distal edits [30].

6. Generate at least five guide RNAs (gRNAs), prioritising proximity to the intended edit. Design corresponding single-stranded oligodeoxynucleotide (ssODN) donor templates (for HDR experiments) in both targeting and non-targeting strand orientations (Fig. 1B and C). Create a schematic overview of the editing strategy in Benchling (Fig. 2A).

7. Particularly if it is not possible to design PAM-modified donor templates with IDT, open the target gene sequence in Benchling and select the “Design HR template”. Introduce the intended edit (Fig. 2B) and paste the gRNA sequence to explore all the PAM-altering alternatives for each design (Fig. 2C). If the edit already alters the PAM, Benchling will return the message “No target sites found for guide (…) and PAM NGG”.
8. If a PAM-altering modification is introduced, validate the variant using different variant analysis software applications, such as MutationTaster2021 (GeneCascade, Charité Universitätsmedizin Berlin), MobiDetails (Montpellier University Hospital) and GeneBe (GeneBe, Medical University of Warsaw), as well as transcript and splicing prediction tools, such as BDGP (Berkeley Drosophila Genome Project, University of California).

### Thawing, Expansion and Freezing of iPSCs

Timing: 1–2 weeks, in preparation of electroporation.

### Before you Start

Perform weekly Mycoplasma testing using the MycoAlert Mycoplasma Detection Kit (Lonza) or a PCR-based method. The optimal incubation times for 0.5 mM EDTA and Accutase treatments should be empirically determined, as they may vary depending on iPSC line and culture conditions used. Rho-associated coiled-coil containing protein kinase (ROCK) inhibitor Y-27632 dihydrochloride can be used at a final concentration of 10 micromolar (µM) as an alternative to CultureCEPT Supplement.

## Procedure

### Thawing and Plating iPSCs

Due to the ~ 18-hour attachment period, we start the procedure in the afternoon (16:00) to allow the first medium change at 10:00.

1. Prepare recombinant human vitronectin-N (VTN)-coated 6-well plates, according to the manufacturer’s recommendations. If using plates stored at 4 °C, allow them to equilibrate at room temperature (RT) for 1 h.
2. Prepare Complete Essential 8 (E8) Medium as described in the supplementary information (“Preparation of cell culture media and buffers”, page 2) and pre-warm it to RT. Add CultureCEPT supplement to the pre-warmed medium.
3. Pre-warm a water bath at 37 °C.
4. Thaw the iPSC cryovial in a water bath at 37 °C by gently swirling and rocking every few seconds until only a small ice crystal remains.
5. Transfer the iPSC suspension to a 15 millilitre (mL) tube using a 10 mL serological pipette.

Note: Avoid pipetting up and down, as this may cause mechanical stress and reduce cell viability.

6. Slowly dispense complete E8 medium supplemented with CultureCEPT dropwise to the tube while gently rocking the tube every few seconds, reaching a final volume of 10 mL.
7. Centrifuge the cell suspension at 100 relative centrifugal force (x g) for 3 min at RT.
8. Carefully remove the supernatant, leaving enough residual medium to cover the cell pellet. Gently tap the bottom of the 15 mL tube to dislodge the pellet.
9. Aspirate the VTN-coated plate.
10. Using a 10 mL serological pipette, resuspend the cells by adding 2 mL of complete E8 medium supplemented with CultureCEPT to the pellet and transfer directly to one VTN-coated well.

Note: Avoid pipetting up and down to minimise mechanical stress, which can compromise viability.

11. Rock the plate with gentle side‑to‑side and back‑and‑forth motions to evenly distribute the iPSCs. Incubate at 37 °C, 5% CO_2_ for 18 h.
12. After 18 h, wash the cells with 2 mL/well Complete E8 medium, aspirate and add 2 mL/well Complete E8 medium.
13. After an additional 24 h, replace the medium daily with 2 mL/well of Complete E8 medium. Monitor the iPSCs daily for growth, density/confluence and morphology.

### Passaging and Expansion of iPSCs

When iPSCs have reached 60–75% confluence, perform EDTA-based clump passaging onto VTN-coated 6-well plates. Prepare 0.5 mM ethylenediaminetetraacetic acid (EDTA) in Dulbecco’s phosphate-buffered saline without calcium and without magnesium (DPBS^−/−^) and coat 6-well plates with VTN.

1. Rinse the iPSCs once with 3 mL/well DPBS^-/-^, then incubate with 1 mL/well 0.5 mM EDTA at 37 °C, 5% CO_2_ for 2–3 min. Colonies should still remain attached, but their margins should begin to loosen and appear slightly refractile or translucent.
2. Aspirate the VTN solution from the new 6-well plate.
3. Carefully remove the 0.5 mM EDTA from the iPSC well.
4. For a standard 1:4 split ratio, gently resuspend the cells in approximately 8 mL of Complete E8 medium using a 10 mL serological pipette. Perform a single, gentle circular motion from the centre outward to evenly detach and resuspend the loosened colonies.

Note: Perform only once. Do not re-attempt detachment if some colonies remain attached after the first pass. Additional pipetting or agitation can damage the cells that have already detached. Any colonies that remain firmly attached should be discarded.

5. Transfer the cell suspension to the new VTN-coated 6-well plate by dispensing 1 mL/well into 4 separate wells twice to reach a final volume of 2 mL/well.
6. Gently rock the plate side‑to‑side and back‑and‑forth to ensure even cell distribution. Incubate at 37 °C, 5% CO_2_ for 24 h.
7. After 24 h, rinse with 2 mL/well Complete E8 medium and feed with 2 mL/well Complete E8 medium. Continue with daily medium changes without rinsing until the cells reach 60–75% confluence and are ready for passaging.

### Freezing iPSCs

The iPSCs should be 60–75% confluent at the time of freezing. We recommend culturing the cells in 6-well plates and resuspending each well in 2 mL freezing medium and aliquoting 1 mL/vial, which typically corresponds to 0.8–1‧10^6^ iPSCs per vial when 60–75% confluent.

1. Prepare > 2 mL Complete E8 medium supplemented with 10% DMSO (freezing medium) for each well of iPSC culture to be cryopreserved.
2. Rinse the cells with 3 mL/well DPBS^-/-^ and incubate with 1 mL/well 0.5 mM EDTA at 37 °C, 5% CO_2_ for 2–3 min.
3. Aspirate the 0.5 mM EDTA solution and resuspend the iPSCs with 2 mL of freezing medium using a 10 mL serological pipette.

Note: Do not attempt to recover colonies left behind after the initial resuspension. The cell suspension should be handled only once, as repeated pipetting can cause substantial loss of cell viability.

4. Transfer the cell suspension into cryovials, dispensing 1 mL/vial.
5. Place the cryovials in a freezing container such as a CoolCell and transfer to a −80 °C freezer for 24-48 h before transferring the vials to < −140 °C for long-term cryostorage.

### CRISPR/Cas9 Editing of iPSCs

Timing: 3–4 days.

### Before you Start

Use E8 medium or StemFlex medium without Penicillin-Streptomycin to avoid potential interference with electroporated CRISPR components and editing efficiency.

It is recommended to empirically determine the optimal StemPro Accutase Cell Dissociation Reagent incubation time. Prolonged exposure may result in decreased cell viability and clonal efficiency.

Use only nuclease-, DNase-, DNA- and RNase-free tubes with safe-lock caps and nuclease-free PCR strips. Assemble CRISPR components in nuclease-free PCR strips and use a thermocycler to ensure precise temperature control and stability during critical incubation steps. Prepare the electroporation station (Neon Electroporation System) before beginning the procedure. Ensure that all components are sterile and correctly assembled. Refer to the instrument manual for step-by-step instructions and visual guidance. Although electroporation in this study was performed using the Neon Electroporation System, other electroporation platforms, such as MaxCyte or Lonza Nucleofector systems, may also be used as suitable alternatives. However, the electroporation program, reagents and methods described here were optimised specifically for the Neon platform and cannot be directly transferred to alternative systems without prior platform-specific adaptation and verification.

Start the procedure in the afternoon (16:00) to allow for an 18-hour post-electroporation recovery period, enabling removal and medium change the following morning (10:00).

### Procedure

1. Rinse the iPSCs once with 3 mL/well Complete E8 medium (without (w/o) Penicillin-Streptomycin) supplemented with 10 µM Y-27632, then incubate with 2 mL/well Complete E8 medium (w/o Penicillin-Streptomycin) supplemented with 10 µM Y-27632 for > 1 h at 37 °C, 5% CO_2_.
2. Add 2 mL/well Complete E8 medium (w/o Penicillin-Streptomycin) supplemented with 10 µM Y-27632 and Alt-R HDR Enhancer V2 0.1–1 µM (only if performing HDR experiment) to a new VTN-coated 6-well plate and place in incubator to pre-warm the medium.

Note: We recommend testing multiple Alt-R HDR Enhancer V2 concentrations in parallel, as each condition will be assessed separately for HDR outcome to identify the optimal edited pool for subsequent expansion/clonal isolation. The final concentration should not exceed 1 µM.

3. Resuspend the Alt-R CRISPR-Cas9 crRNA and tracrRNA in nuclease-free Duplex Buffer to a final concentration of 100 µM (10 µL Duplex Buffer/1 nmol).
4. Prepare the crRNA: tracrRNA duplex by mixing the two RNA oligos at equimolar concentrations and add nuclease-free Duplex Buffer to a final concentration of 44 µM:

**Table:** 

Component	5 rxns (µL)	20 rxns (µL)
crRNA (100 µM)	1.1	4.4
tracrRNA (100 µM)	1.1	4.4
Nuclease-free Duplex Buffer	0.3	1.2
Total volume	2.5	10

Note: It is recommended to always prepare a larger volume to improve pipetting accuracy and mixing precision. Avoid pipetting volumes < 1 µL, especially with glycerol-containing reagents. Total volumes of 2.5 and 10 µL will cover 5 and 20 electroporation reactions, respectively.

5. Incubate the crRNA: tracrRNA duplex at 95 °C for 5 min, then allow it to gently cool down at RT for 8–10 min on the bench.
6. Dilute the Alt-R S.p. HiFi Cas9 Nuclease V3 stock (61 µM) to 36.6 µM in Resuspension Buffer R (Neon Electroporation System):

**Table:** 

Component	5 rxns (µL)	20 rxns (µL)
Cas9 Nuclease (61 µM)	1.5	6
Resuspension Buffer R	1	4
Total volume	2.5	10

7. Combine equal volumes of the crRNA: tracrRNA duplex and the diluted Cas9 to form the RNP complex:

**Table:** 

Component	5 rxns (µL)	20 rxns (µL)
Cas9 Nuclease (36 µM)	2.5	10
crRNA: tracrRNA duplex	2.5	10
Total volume	5	20

8. Incubate the RNP complex at RT for 20 min.
9. Resuspend the Alt-R HDR Donor Oligo in nuclease-free Duplex Buffer to prepare a 100 µM stock solution (10 µL Duplex Buffer/1 nmol).
10. Dilute the Alt-R HDR Donor Oligo 100 µM stock to 10.8 µM in Resuspension Buffer R:

**Table:** 

Component	5 rxns (µL)	20 rxns (µL)
HDR Donor Oligo (100 µM)	1.2	4.8
Resuspension Buffer R	9.9	39.6
Total volume	11.1	44.4

Note: If HDR is not performed, steps 9 and 10 are omitted.

11. Wash the iPSCs twice with DPBS^-/-^ supplemented with 10 µM Y-27632, then incubate with 1 mL/well StemPro Accutase Cell Dissociation Reagent supplemented with 10 µM Y-27632 for 9–10 min at 37 °C, 5% CO_2_.
12. Gently resuspend the iPSCs with 3 mL/well Complete E8 medium (w/o Penicillin-Streptomycin) supplemented with 10 µM Y-27632. Filter the suspension through a 40 μm cell strainer and count the cells.
13. Centrifuge the cell suspension at 100 x g for 3 min. Resuspend the pellet in Resuspension Buffer R to 2.22‧10^7^ cells/mL (equivalent to 2‧10^5^ cells/reaction).
14. Turn on the Neon Electroporation System and enter the following settings:
  Voltage = 1400 V.
  Pulse width = 20 ms.
  Pulse number = 1 pulse.

15. Insert the Neon Tube into the Neon Station and then add 3 mL/tube Electrolytic Buffer.
16. Prepare the CRISPR reaction mix as follows:

**Table:** 

Component	Per rxn (µL)
Alt-R HDR Donor Oligo (10.8 µM)	2
RNP complexes	1
iPSCs (2.22‧10^7^ cells/mL)	9
Total volume	12

Note: Ensure that the electroporation mixture is homogeneously mixed prior to electroporation. Pipette gently and avoid introducing air bubbles. Avoid keeping the iPSCs in Resuspension Buffer R for extended periods, as this may increase cellular stress and reduce viability. If performing multiple electroporation reactions, prepare all CRISPR components in advance and add the iPSCs in a streamlined manner to minimise delays prior to electroporation. If HDR is not performed, replace the HDR Donor Oligo with equal volumes of Resuspension Buffer R.

Note: In the final electroporation mixture, the gRNA duplex, Cas9 nuclease and donor oligo were present at an approximate molar ratio of 1.20:1:1.18 (gRNA duplex: Cas9 nuclease: donor).

17. Use the 10 µL Neon Pipette to collect the electroporation mixture and insert it into the Neon Station.
18. Perform electroporation.
19. Remove the Neon Pipette and dispense the iPSCs directly into one well of the VTN-coated 6-well plate containing pre-warmed E8 medium.

Note: Do not reuse the same Neon Tube across different RNP complexes and/or iPSC lines to prevent cross-contamination.

20. Incubate the cells at 37 °C, 5% CO_2_ for 18 h.
21. After 18 h, replace the medium with fresh Complete E8 medium (w/o Penicillin-Streptomycin) to remove Y-27632 and Enhancer V2.
22. Continue replacing the medium every 24 h with Complete E8 medium until the cells reach 60–75% confluence, at which point they can be passaged and expanded.

Note: Do not use iPSCs for downstream applications < 72 h post-electroporation.

### Screening of NHEJ and HDR in CRISPR-Edited iPSC Pools

Timing: 2 days.

### Before you Start

**Fig. 3:**
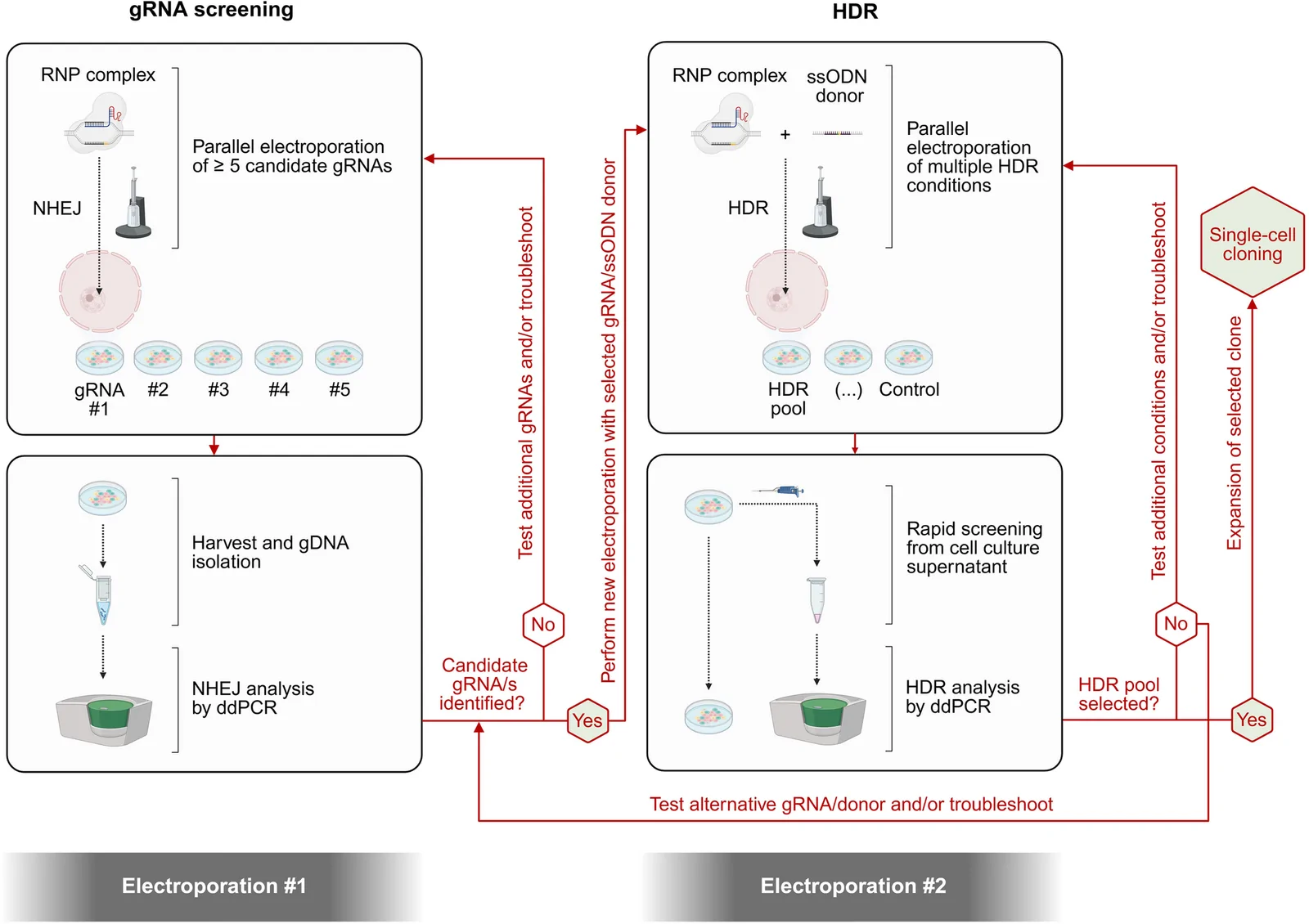
Sequential decision-making workflow for gRNA screening and selection, HDR analysis and optimisation, and single-cell cloning in CRISPR-edited iPSCs. Candidate gRNAs are first screened in parallel by RNP electroporation in the absence of a ssODN donor to identify the most suitable guide for subsequent HDR experiments. Subsequently, in a second electroporation, the selected gRNA is combined with an ssODN donor template and evaluated across multiple conditions in parallel, followed by rapid HDR analysis to identify the most suitable edited pool for further progression. This strategy enables both qualitative and quantitative comparison of editing outcomes and cell recovery before committing to labour-intensive and resource-demanding clonal isolation

**Fig. 4:**
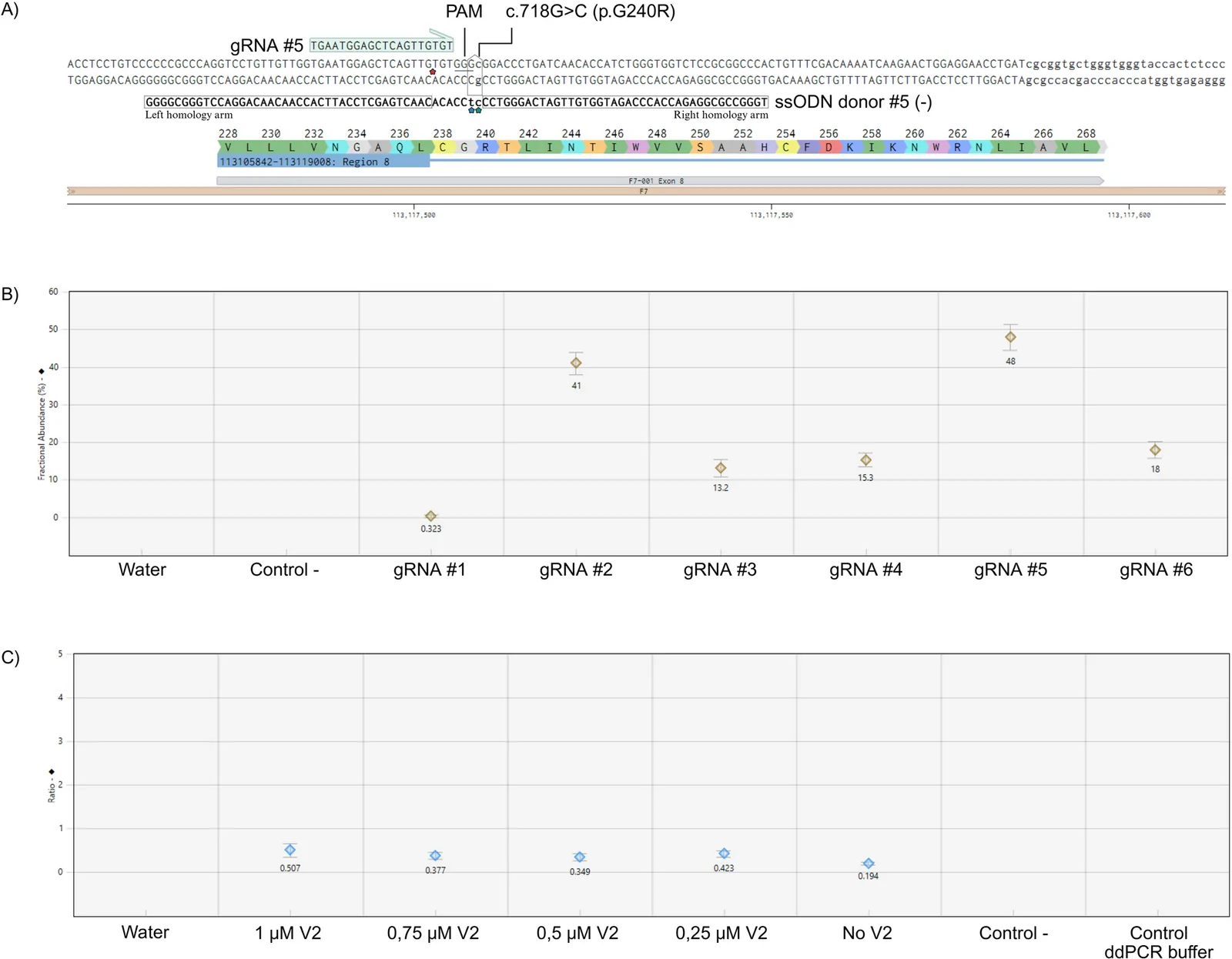
NHEJ and HDR detection by ddPCR. (**A**) Overview of the selected HDR-based correction strategy (gRNA #5 and ssODN donor #5 (-)). The cut site is indicated by a red star, while the synonymous PAM-altering mutation and the therapeutic correction are indicated by a blue and green star, respectively. (**B**) ddPCR NHEJ Genome Edit Detection Assays of the different gRNAs screened in Roman et al., 2026[29]. On-target efficiency is displayed as fractional abundance (%), calculated as NHEJ-edited alleles/(WT + NHEJ-edited alleles), i.e. edited alleles/total alleles. (**C**) ddPCR Homology Directed Repair Genome Edit Detection (HDR GED) assay for interrogating the HDR outcome at different concentrations of Alt-R HDR Enhancer V2, from Roman et al., 2026[29]. The ratio provided is the number of HDR alleles/total number of alleles, and the percentage of total HDR efficiency can be obtained as ratio‧100

Screening for NHEJ editing in CRISPR-edited iPSC pools is a useful preliminary step to identify the suitable gRNA candidates for subsequent HDR experiments (Fig. 3). In Roman et al., 2026 [29], this approach was applied to six candidate gRNAs (Fig. 4B) to identify the most suitable guide for subsequent HDR editing (Fig. 4C). Importantly, the NHEJ screening experiment is performed in the absence of a donor DNA template to assess guide-dependent cleavage activity independently of HDR. A minimum of five candidate sgRNAs is recommended for reliable comparison and guide selection. Therefore, NHEJ screening is performed on CRISPR-edited iPSC pools that are treated as terminal samples and gDNA is isolated from a conventional cell pellet. By contrast, early HDR analysis can be performed on recovering electroporated iPSC pools simultaneously across multiple experimental conditions at once while the cells remain in culture, enabling only selected pools to be advanced for expansion and downstream single-cell cloning. Because a conventional cell pellet is unavailable until a pool has been passaged, gDNA can be prepared from cellular material recovered from the culture supernatant at least 72 h after electroporation.

## Procedure

### NHEJ Detection by ddPCR

1. Design the customised NHEJ detection assay by opening the Assay Design Engine Tools (BioRad) and selecting “NHEJ edit”. When an NHEJ assay is designed for a target sequence containing a patient-specific variant that is not present in the Homo sapiens reference genome, as is the case in gene correction experiments, select “The sequence being edited is a variation of the Homo sapiens reference genome” and “This context sequence > Replaces the homologous sequence in the background genome”.

Note: The NHEJ assay can also be configured to detect NHEJ events in HDR experiments by selecting “Yes” under “Is there a donor present in the reaction?” during assay design, if applicable.

2. Access the gene sequence in Benchling and copy a region spanning ≥ 1500 bp upstream and downstream of the predicted cut site. Paste the full sequence into the “Context sequence” field and annotate the predicted cut site using a caret symbol (^) (e.g., CCA^TTT). Generate the ddPCR NHEJ detection assay.

Note: If the NHEJ assay is not designable due to sequence complexity, contact the manufacturer’s technical support for assistance or proceed with a T7EI assay.

3. After ≥ 72 h from electroporation, isolate and quantify the genomic DNA (gDNA) using the DNeasy Blood & Tissue Kit.
4. Thaw all reagents, mix thoroughly and spin down. Set up the following reaction:

**Table:** 

Component	Per rxn (µL)
ddPCR Supermix for Probes (No dUTP) (2X)	11
NHEJ Genome Editing Detection Assay (20X)	1.1
(Optional: Diluted restriction enzyme)	Variable (2–5 U/reaction)
gDNA template	Variable (20 ng)
Nuclease-free water	To 22

Note: For optimal performance, particularly when using > 60 ng/reaction of DNA, long amplicons (> 150 bp), or GC-rich targets, restriction digestion is recommended. DNA fragmentation enables optimal accuracy by separating tandem gene copies, reducing sample viscosity, and improving template accessibility.

Dilute the restriction enzyme according to the manufacturer’s instructions and add 1 µL directly to the reaction mix.

5. Mix thoroughly by vortexing and spin down, then incubate the reaction at RT for 3 min.
6. Load 20 µL of the reaction mix into a sample well of a DG8 Cartridge and fill any unused wells with 20 µL Buffer Control, pre-diluted in nuclease-free water to a final 1X working concentration. Add 70 µL Droplet Generation Oil to all 8 wells.
7. Attach the DG8 Gasket across the DG8 Cartridge and place it into the QX200 Droplet Generator (BioRad) to start droplet generation.
8. Carefully transfer 40 µL droplets into a ddPCR 96-well plate and cover with the seal foil. Seal with a PX1 PCR Plate Sealer and transfer to thermocycler to perform the following PCR program with the lid heated to 105 °C:

**Table:** 

Cycle step	Temperature	Time	Ramp rate	Cycles
Enzyme activation	95 °C	10 min	2 °C/sec	1
Denaturation	94 °C	30 s		40
Annealing/extension	55 °C	Variable		
Enzyme deactivation	98 °C	10 min		1
Hold	4 °C	∞	1 °C/sec	1

Note: Begin with an annealing/extension time of 3 min. If optimisation is needed, increase annealing/extension time to 6 min. If results are satisfactory, further optimise by gradually reducing the annealing/extension time in 1-minute intervals to identify the minimal optimal annealing/extension time.

9. Open QuantaSoft Software (BioRad) and start a new plate experiment. Select:
  Experiment type: ABS.
  Supermix: ddPCR Supermix for Probes (No dUTP).
  Target 1 (Ch1): FAM.
  Target 2 (Ch2): HEX.

10. Insert the plate into the QX200 Droplet Reader, then start the droplet analysis after selecting the appropriate dye set and run options.
11. Open the droplet reading results in QX Manager Software Standard Edition and designate the experiment as “Drop off (DOF)”. In the 2D amplitude, verify that the FAM^+^ (blue), FAM^+^/HEX^+^ (orange) and FAM^-^/HEX^-^ (grey) clusters are correctly annotated and separated; otherwise proceed with manual thresholding.
12. Visualise the fractional abundance (%) (Fig. 4B), which is defined as the ratio between edited alleles and total alleles (NHEJ-edited and wild-type alleles).

### HDR Detection by ddPCR

1. Open the Assay Design Engine Tool (BioRad) and select “HDR edit”. If performing an HDR-mediated correction, select “The sequence being edited is a variation of the Homo sapiens reference genome” and “This context sequence > Replaces the homologous sequence in the background genome”.
2. On Benchling, access the full genomic sequence and copy a large segment, ideally spanning ~ 1000 bp upstream and downstream of the designed edit. Paste the sequence in the “Context sequence” field.
3. Paste the designed donor template sequence into the “Donor Sequence” field. Submit to generate the ddPCR HDR Genome Edit Detection Assay.
4. At the bottom of the assay detail page, identify the recommended reference assay and open its details.

Note: If needed, identify a restriction enzyme that is compatible with both the customised HDR Genome Edit Detection Assay and the reference assay.

5. After ≥ 72 h following electroporation, collect cell culture supernatant without disturbing the CRISPR-edited iPSC pools and continue with the normal feeding schedule. Isolate gDNA using Phire Animal Tissue Direct PCR Kit (as described in “Screening and expansion of the clonal iPSCs” section) for early HDR detection across multiple experimental conditions. Alternatively, once the edited iPSC pools have been passaged, genomic DNA can be isolated from a conventional cell pellet using the DNeasy Blood & Tissue Kit.
6. Thaw all the ddPCR components, mix thoroughly, and spin down briefly. Assemble the reaction as follows:

**Table:** 

Component	Volume (µL)
ddPCR Supermix for Probes (No dUTP) (2X)	11
HDR Genome Editing Detection Assay (20X)	1.1
HDR Reference Assay (20X)	1.1
(Optional: Diluted restriction enzyme)	Variable (2–5 U/reaction)
gDNA template	Variable (20 ng)
Nuclease-free water	To 22

7. Mix thoroughly by vortexing and spin down, then incubate the reaction at RT for 3 min. Proceed as described previously in steps 6–10 of “NHEJ detection by ddPCR” (pages 14–15).
8. Open the results in QX Manager Software. In the 2D amplitude verify that the FAM^+^/HEX^-^ (blue), FAM^+^/HEX^+^ (orange), FAM^-^/HEX^+^ (green) and FAM^-^/HEX^-^ (grey) clusters are correctly annotated and separated; otherwise proceed with manual thresholding.
9. Select “Ratio” and calculate the percentage of HDR-edited alleles (Fig. 4C), i.e., ratio of edited copies to total copies, as follows: HDR events = ratio‧100.

### Sanger Sequencing

1. Retrieve the target gene sequence from Benchling and design a PCR strategy for Sanger sequencing, favouring the generation of an amplicon with ≥ 700 bp spanning upstream and downstream of the expected cut site.
2. Assemble the PCR reaction using the following steps:

**Table:** 

Component	Volume (µL)
AmpliTaq Gold 360 Master Mix (2X)	12.5
Primer forward (10 µM)	1
Primer reverse (10 µM)	1
(Optional: 360 GC Enhancer)	(Variable)
gDNA template	Variable (10 ng)
Nuclease-free water	To 25

3. Run the PCR using the following thermocycling conditions:

**Table:** 

Cycle step	Temperature	Time	Cycles
Initial denaturation	98 °C	30 s	1
Denaturation	98 °C	30 s	35
Annealing	Variable	30 s	
Extension	72 °C	Variable	
Final extension	72 °C	7 min	1
Hold	10 °C	∞

4. Verify PCR by agarose gel electrophoresis and submit the PCR product for Sanger sequencing.

### Single-Cell Cloning of iPSCs

Timing: 1–2 weeks.

### Before Proceeding

We recommend passaging the selected electroporated pools at least once before performing single-cell cloning. Ensure iPSCs are in the exponential growth phase and at 60–75% confluence to maximise cloning efficiency and downstream clonal quality. Avoid overconfluent cultures, which may reduce cloning efficiency and clonal outgrowth after dispensing. Start the procedure in the afternoon (16:00–19.00, day 0) to allow for the clonal iPSCs to remain untouched until the early morning of day 3 (64 h). We recommend limiting ROCKi exposure (CultureCEPT Supplement or 10 µM Y-27632) to the minimum necessary and avoiding extended incubations. Y-27632 can be used at a final concentration of 10 µM as an alternative to CultureCEPT Supplement.

As described previously, iPSCs were routinely maintained in E8 medium. However, for single-cell cloning and the early post-sorting recovery phase, iPSCs were cultured in StemFlex medium supplemented with CultureCEPT, as this condition has been reported to provide superior cytoprotection, survival and clonal expansion under high-stress culture conditions [31, 34]

Pre-warm only the required volume of StemFlex medium by gradually equilibrating it to RT, according to the manufacturer’s instructions. Only when explicitly indicated in the procedure, further warm the medium from RT to 37 °C.

### Procedure

1. Prepare VTN-coated 96-well plates. After coating, aspirate the VTN solution and replace it with 100 µL/well of complete StemFlex medium supplemented with CultureCEPT Supplement. Pre-warm the plates at 37 °C, 5% CO_2_ until ready to use.

Note: The concentration of VTN coating can be increased to 1 µg/cm^2^ to further improve single-cell cloning efficiency by promoting stronger cell attachment and survival.

2. Pre-incubate the selected iPSC pool, cultured in a 6-well plate, with 2 mL/well complete StemFlex medium supplemented with CultureCEPT Supplement. Pre-warm the medium to 37 °C before use, then incubate the cells for > 1 h at 37 °C, 5% CO_2_.
3. Gently rinse the iPSCs twice with DPBS^-/-^ supplemented with CultureCEPT Supplement, then incubate with 1 mL/well StemPro Accutase Cell Dissociation Reagent supplemented with CultureCEPT Supplement for ≤ 10 min at 37 °C, 5% CO_2_.
4. Gently resuspend iPSCs in ≥ 3 mL/well of StemFlex medium supplemented with CultureCEPT Supplement. Filter the suspension through a 40 μm cell strainer and count cells.
5. Proceed to single-cell cloning.

### Single-Cell Cloning Using UP.SIGHT

**Fig. 5:**
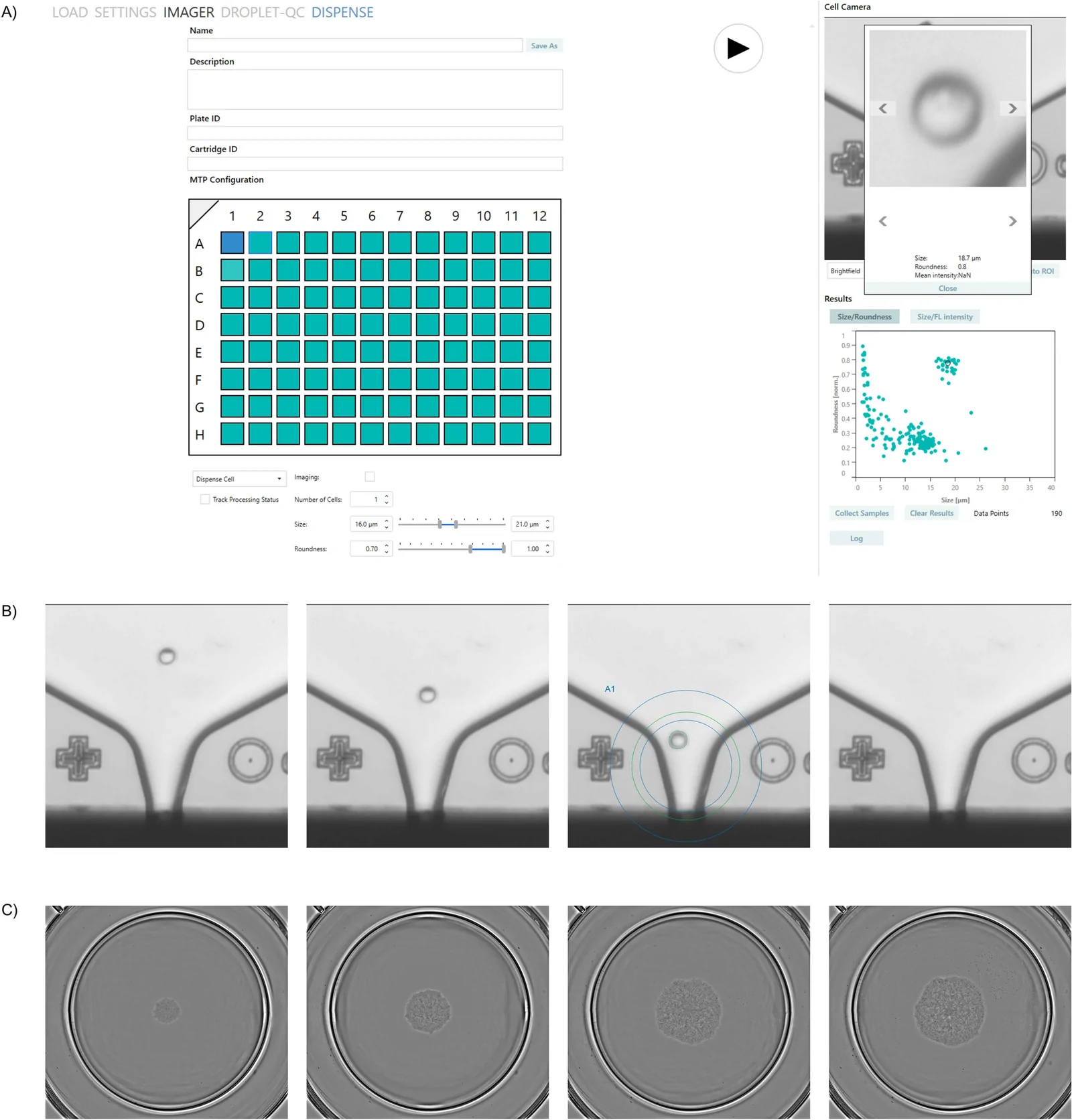
Single-cell iPSC cloning using UP.SIGHT (Cytena). (**A**) Plate view before dispensing (left) and results from the sample collection and analysis prior to single-cell dispensing (right). (**B**) Nozzle imaging of a single cell before, during and after dispensing (from left to right). (**C**) Plate imaging of a single cell-derived iPSC clone at 4, 6, 8 and 10 days (left to right) after single-cell deposition

Note: Avoid any unnecessary movement or exposure of the plate during this initial incubation, as this period is critical for clonal cell attachment and recovery.

a Centrifuge the filtered cell suspension at 100 x g for 3 minutes. Carefully aspirate the supernatant, gently tap the tube to loosen the pellet and resuspend the iPSCs at 5‧10^5^ cells/mL in DPBS^-/-^ supplemented with CultureCEPT Supplement.
b Transfer 80 µL of the cell suspension (4‧10^4^ iPSCs) into an EASY.ON Cartridge 40x40 (Cytena). Load the EASY.ON Cartridge into the dispensing slot and ensure that the agitation tubing is properly connected to the agitation adapter. Avoid introducing air bubbles into the cartridge during loading.
c Place the pre-warmed VTN-coated 96-well plate in the UP.SIGHT plate adapter.
  Note: Before proceeding, verify that “agitation”, “idle shooting”,“dispensing” and “deionization” functions are enabled.
d Adjust the nozzle position by setting the correct focus, lateral and vertical position. Follow the manufacturer's guidance to calibrate the system. Complete the droplet quality control (droplet-QC) step before continuing. Repeat the droplet-QC for each new dispensing run, and for every 96-well plate, to ensure dispensing accuracy and system consistency.
e Press “Collect samples” to allow the system to analyse the iPSC population based on size (µm) and roundness (Fig. 5 A). We recommend the following settings under the “MTP configuration”:
  Minimum size (µm) = 15
  Maximum size (µm) = 22–23
  Minimum roundness = 0.65–0.7
  Maximum roundness = 1
f Dispense 1 cell/well (Fig. 5 A and B).
g Once dispensing is complete (16.00 −19.00 pm, day 0), incubate the plate at 37 °C, 5% CO_2_ for 64 hours without disturbance (Fig. 5 C).

###  Single-Cell Cloning Using Dispencell-S3

- Centrifuge the filtered cell suspension at 100 x g for 3 min. Carefully aspirate the supernatant, gently tap the tube to loosen the pellet and resuspend the iPSCs at 2‧10^5^ cells/mL with StemFlex medium supplemented with CultureCEPT Supplement.
- Mix 15 µL of cell suspension (3’000 cells) in the DispenMe vial (135 µL buffer) and gently pipette to resuspend without introducing bubbles.

Note: ART Wide Bore Filtered Pipette Tips are recommended to facilitate efficient mixing of the cell suspension without introducing air bubbles.

c. Load the DispenMe vial into the sample slot of the Dispencell-S3 platform. Fill the washing unit with 25 mL DPBS^-/-^ and place it in the designated wash slot. Set the system loading time as follows:
  96-well plate: 60 seconds
  2‧96-well plates: 120 seconds

d. Initiate histogram generation and perform a quick quality control (20 events, 30 s analysis). Confirm the following readout parameters:
  Pressure = −150/−100 mbar
  Impedance = 30’000–50’000 ohms

e. Review the estimated dispensing metrics:
  Tcc (time between two cell events)
  PFR (% single cells/well)
  Total time of single-cell dispensing

Note: Aim for a Tcc of 2–5 s. Lower Tcc values result in faster dispensing but reduce accuracy and sensitivity.

f. Run the automated wash cycle and monitor the system “live values”.

Note: Pressure during this step should be 10–20 mbar. The debris-cell impedance threshold typically ranges from 200 to 400 ohms.

**Fig. 6:**
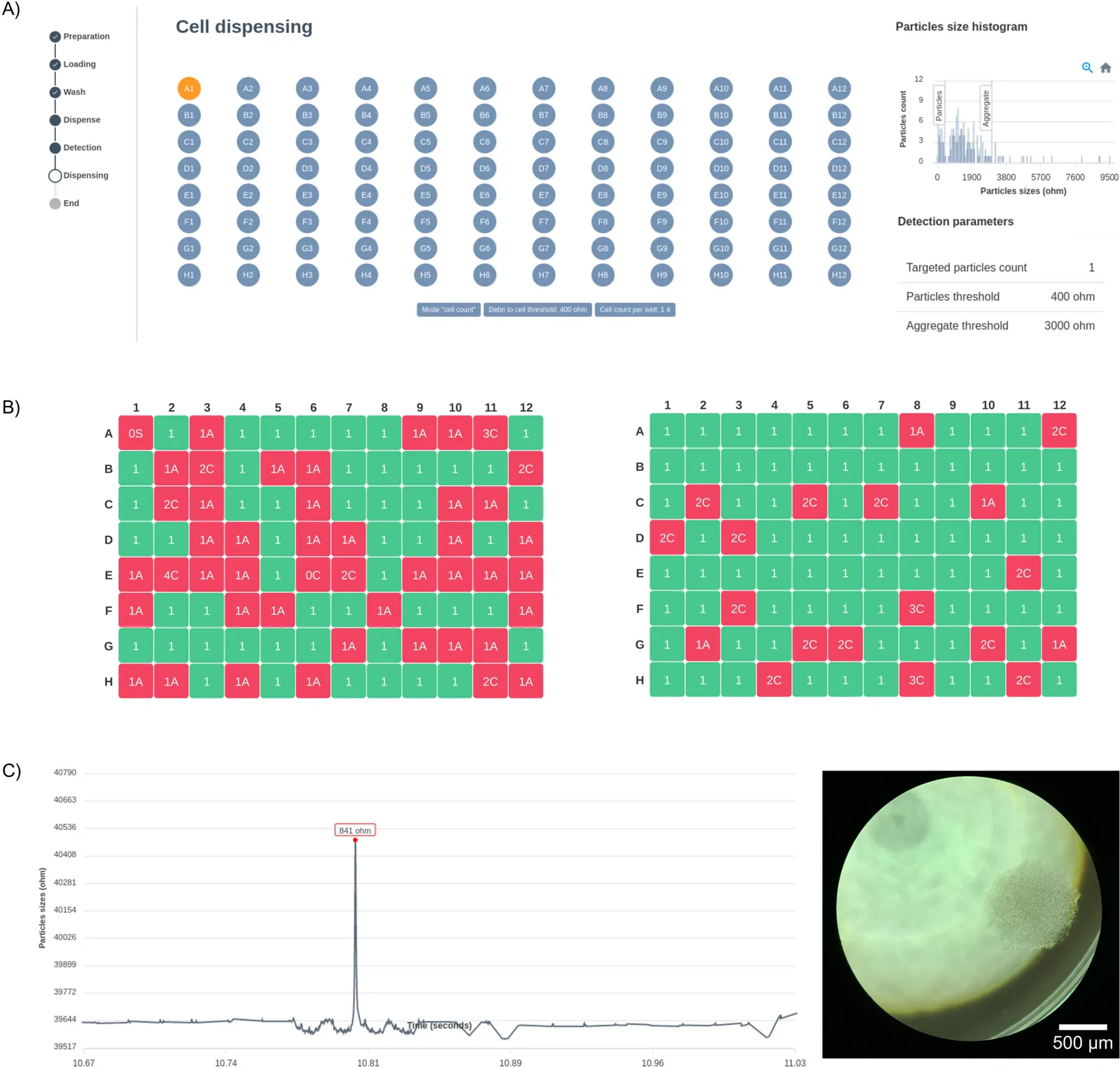
Single-cell iPSC cloning using Dispencell-S3 (SEED Biosciences). (**A**) Plate view before dispensing (left), and common particle and aggregate threshold values for iPSC dispensing (right). (B, left) Representative particle analysis report showing a plate filling rate (PFR) of 52% after single-cell dispensing with the Dispencell-S3 (SEED Biosciences). Discarded wells are highlighted in red. Aggregates, defined by an impedance particle threshold > 3000 ohms, are labelled “A”. Wells with more than one dispensed cell are labelled “C”, indicated by the corresponding cell number. Wells showing signal saturation during recording are labelled “S”. (B, right) Representative particle analysis report with a PFR of 80%. (C, left) Example histogram plot of a single-cell event. The measured impedance peak for the single cell (841 ohms) is indicated above the signal. The x-axis represents time (seconds) and the y-axis represents impedance (ohms). (C, right) Example of a clonal iPSC colony 8 days after single-cell dispensing. ECHO Revolve RVL-100-M (500 μm scale bar)

g. Place the VTN-coated 96-well plate, pre-warmed at 37 °C, 5% CO₂, into the plate scaffold. Select “Cell Count” as the dispense mode to deliver precisely 1 cell/well. Check the “Cell Detection Parameters” to ensure appropriate particle and aggregate thresholds (Fig. 6A).

h. When dispensing is complete (16.00–19.00 pm, day 0) (Fig. 6B and C), incubate the iPSCs at 37 °C, 5% CO_2_ for 64 h.

Note: Avoid unnecessary movement or exposure of the plate during this initial incubation, as this period is critical for clonal cell attachment and recovery.

Note: Identify wells for exclusion by cross-referencing the DispenCell Analyzer plate map with the dispensing histogram (Fig. 6B and C). Discard wells that show uncertain histogram plots where the single-cell event cannot be unambiguously assigned, aggregates at seeding, more than one cell per well, or multiple colonies during outgrowth.

6. After 64 h (day 3, morning), gently add 125 µL/well pre-warm complete StemFlex medium on top of the existing 100 µL/well. Pre-warm the medium to 37 °C before use.

Note: This step dilutes the CultureCEPT Supplement while maintaining continuous recovery support with complete StemFlex medium. It is possible to try reducing the first incubation step to 48 h; however, this alternative timing was not experimentally tested in the present study.

7. After an additional 48 h (day 5, morning), remove 150 µL/well of medium and replace with 160 µL/well fresh complete StemFlex medium. Ensure that a layer of medium continues to cover the bottom surface of each well.
8. After an additional 48 h (day 7, morning), remove 150 µL/well of medium and replace with 160 µL/well fresh complete StemFlex medium.

Note: Daily medium changes can be initiated from day 6; however, this alternative timing was not experimentally tested in the present study and should be considered based on the characteristics of each individual iPSC line.

9. Continue daily medium changes (day 8, morning) until colony outgrowth exceeds 30% confluence. Around day 8 or later, perform plate imaging of the clonal colonies.

Note: Remove medium from wells that are empty or that contain multiple cells or colonies, as these wells are not suitable for downstream applications.

10. Transfer iPSC colonies ready for passaging (30–75% confluence, ~day 10) to fresh VTN-coated 24-well plates pre-filled with 300 µL complete StemFlex supplemented with CultureCEPT Supplement. Gently rinse with 200 µL/well DPBS^-/-^ and incubate the selected clonal iPSCs with 65 µL/well StemPro Accutase Cell Dissociation Reagent at 37 °C, 5% CO_2_ for 5 min.

Note: EDTA may be used as an alternative to StemPro Accutase Cell Dissociation Reagent.

11. Gently aspirate the StemPro Accutase Cell Dissociation Reagent and resuspend the colonies in 200 µL complete StemFlex supplemented with CultureCEPT Supplement. Transfer the suspension to a VTN-coated 24-well plate (pre-filled with 300 µL complete StemFlex supplemented with CultureCEPT Supplement). Incubate at 37 °C, 5% CO_2_ for 24 h.
12. Replace the culture medium daily with 500 µL complete StemFlex medium until the clonal iPSCs reach 60–75% confluence.
13. Optional: during this expansion phase in a 24-well plate, it is possible to collect the cell culture supernatant and rapidly perform the first-line genetic screening by Sanger sequencing. Proceed to the next chapter “Screening and expansion of the clonal iPSCs”, step 1.

Note: We recommend performing Sanger sequencing at this stage to selectively continue expanding only the desired clonal iPSCs, thereby optimising time and resources. If needed, two consecutive supernatant collections can be pooled to reach a starting volume of 1 mL.

### Screening and Expansion of the Clonal iPSCs

Timing: 1 week.

### Before Proceeding

Perform Sanger sequencing during the expansion phase in 24-well plates to selectively expand only the desired clonal iPSC lines. This targeted approach helps optimise time and resources by avoiding the need to freeze and store large numbers of low-quality or undesired clones. Instead of freezing the entire clonal population prematurely, we recommend expanding only selected, sequence-verified clones and generating larger, high-quality cryopreserved batches after confirmation. This reduces unnecessary freeze-thaw cycles and preserves the long-term viability and function of the best-performing iPSC clones.

### Procedure

1. Collect the cell supernatant and centrifuge at 500 x g for 5 min.
2. Carefully aspirate the supernatant and resuspend the pellet in 20 µL dilution buffer and 0.5 µL DNA Release Additive (Phire Animal Tissue Direct PCR Kit). Mix well by briefly vortexing and spin down.

Note: If a clear pellet is not visible, leave 30 µL residual supernatant to reduce the risk of sample loss. However, this will result in lower final DNA concentration.

**Fig. 7:**
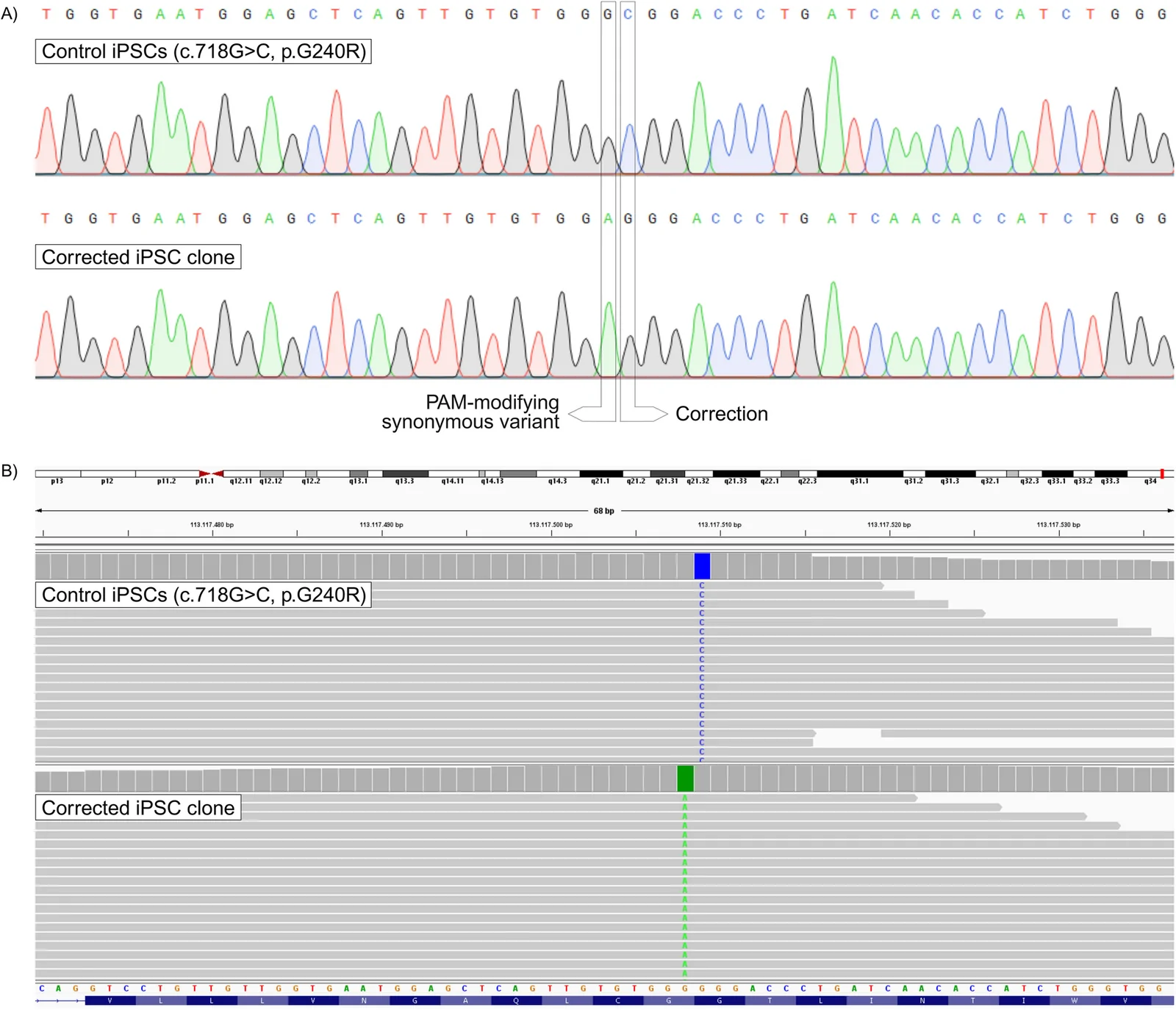
Genetic characterization of a gene-corrected, single cell-derived iPSC clone. (**A**) Sanger sequencing view of the gRNA #5/ssODN donor-targeted locus in exon 8 of *F7*. The c.718G > C, p.G240R control is shown at the top and a homozygous corrected iPSC clone at the bottom. Generated with SnapGene Viewer 7.2.1. (**B**) Integrative Genomics Viewer (IGV; igv.org) view from the Illumina Clinical Research Exome-CRE V4 analysis. Sequencing reads are presented in grey, the GRCh38/hg38 reference sequence is located at the top under the chromosomal coordinates. c.718G > C, p.G240R control (top) and a homozygous, corrected iPSC clone (bottom). Adapted from Roman et al., 2026 [29].

3. Incubate the tubes for 2–5 min at room temperature, then immediately transfer to the pre-warmed heat block at 98 °C for 2 min.
4. Briefly spin down the tubes to collect condensation and proceed directly with PCR amplification and gel electrophoresis, using the primers and conditions optimised in the “Screening of NHEJ and HDR in CRISPR-edited iPSC pools” chapter “Sanger sequencing”.
5. Validate the PCR product by agarose gel electrophoresis and perform Sanger sequencing (Fig. 7A).

6. Based on the sequencing results, identify the clonal iPSCs to culture until 60–75% confluence is reached.

Note: The iPSCs can be re-adapted from complete StemFlex to complete E8 medium following the manufacturer’s instructions.

7. At 60–75% confluence, perform clump passaging. Gently rinse each well of the 24-well plate with 500 µL of DPBS-/-, then incubate with 250 µL/well of 0.5 mM EDTA at 37 °C, 5% CO₂ for 2–3 min. Prepare VTN-coated 6-well plates and pre-fill each well with 1 mL/well of E8 medium, if re-adaptation from StemFlex to E8 medium has been performed.
8. Aspirate the 0.5 mM EDTA solution. Gently resuspend the clonal iPSCs in 1 mL/well of complete E8 medium. Transfer the suspension to the pre-filled 6-well plate (1 mL/well) and incubate at 37 °C, 5% CO₂ for 24 h.
9. Once sufficient cell numbers are reached, generate cryopreservation batches of the selected iPSC clones using the optimised freezing conditions detailed in “Freezing iPSCs” in the “Thawing, expansion and freezing of iPSCs” chapter.
10. Perform downstream genetic characterisation of selected clones using progressively more refined analyses, as follows:

- Isolation of gDNA from cell culture supernatant using Phire Animal Tissue Direct PCR Kit and analysis by Sanger sequencing.

Note: This step is used for rapid preliminary clonal screening when iPSC clones cannot be harvested and a conventional cell pellet is unavailable.

b. Isolation of gDNA from cell pellet using DNeasy Blood & Tissue Kit and analysis by Sanger sequencing.
c. Isolation of high-quality, high-molecular weight gDNA with Blood & Cell Culture DNA Mini Kit and next generation sequencing (NGS) with Clinical Research Exome-CRE V4 (30x) and/or INVIEW Human Exome (100x) whole exome sequencing (WES) (Fig. 7B).
d. Optional: CIRCLE-seq (or GUIDE-seq) [32–37].
e. Optional: Conventional G-banding with targeted ddPCR/qPCR-based assays and SNP-array/Chromosomal Microarray Analysis (CMA)-based copy-number profiling, such as iCS-Digital PSC and iCS-digital Aneuploidy (StemGenomics), and KaryoStat (Thermo Fisher Scientific), respectively.

Note: Before proceeding with advanced genome-wide analyses such as WES or whole-genome sequencing (WGS) and selecting the clonal iPSC line for downstream experiments, we recommend using the functional integrity and differentiation potential of the edited clonal iPSC line as eligibility criteria. In our workflow, directed organoid differentiation is performed to compare differentiation efficiency across candidate clones before selecting the final line. Whole-exome sequencing (WES) of the selected clone was subsequently performed to confirm clonality and to rule out off-target events.

## Discussion

This protocol outlines the gene editing and clonal expansion of iPSCs used to correct genetic mutations in patient-derived cells from individuals with congenital coagulation disorders [29]. This approach facilitates robust gene editing/correction across multiple loci in both healthy and patient iPSC lines, overcoming the technical challenges typically associated with genome editing.

Achieving high frequencies of HDR without compromising cell quality in human iPSCs remains a challenge due to competing DNA repair pathways and the intrinsic sensitivity of these cells to stress, particularly to dissociation- and p53-induced apoptosis [18, 38, 39]. The CRISPR-Cas9 system mediates targeted genome editing through a double-strand breaks (DSB) at a specified genomic locus, which is then repaired by either the error-prone NHEJ or the HDR pathway. Two major factors hinder efficient homozygous HDR in human iPSCs: the restricted timing of HDR within the cell cycle and the activation of p53 pathways following Cas9-induced DSBs. [18, 38]. Although next-generation genome-editing technologies such as prime and base editing are emerging [40–42], ex vivo electroporation of CRISPR-Cas9 RNPs remains, to date, the only genome-editing approach to receive regulatory approval for clinical use, specifically for the treatment of sickle cell disease and β-thalassemia [43]. Base editing and prime editing are precision-editing platforms that enable sequence modifications without creating a classical DSB [44–47]. Base editors generally consist of a catalytically impaired or nickase Cas9 fused to a deaminase enzyme. Upon target recognition, the Cas9 generates an R-loop that exposes single-stranded DNA, allowing the tethered deaminase to catalyse base conversion [40, 44–46, 48, 49]. Prime editing instead couples Cas9 nickase (nCas9) to a reverse transcriptase and uses an extended prime-editing guide RNA (pegRNA) that both specifies the target site and encodes the desired edit, without requiring a separate donor template [47]. The RNP complexes comprise the recombinant Alt-R S.p. HiFi Cas9 Nuclease V3 and a functional gRNA duplex, which is assembled by annealing synthetic Alt-R crRNA and tracrRNA. The use of RNP complexes, transiently delivered via the Neon electroporation system (Thermo Fisher Scientific), ensures the nuclease is catalytically active immediately upon cellular entry, coinciding with the narrow window of HDR competence, before being rapidly degraded. This transient presence maximises on-target editing while minimising the risk of off-target events [26, 27]. In contrast, vector-based CRISPR editing relies on the host cell's transcriptional and translational machinery for component synthesis. This results in prolonged nuclease activity, often exceeding 72 hours, which increases the risk of off-target effects and introduces experimental variability due to fluctuating expression levels [50]. In this context, the choice of transfection or delivery platform is itself a major determinant of cell survival and genome-editing performance in human iPSCs. Electroporation transiently permeabilizes the cell membrane with a short electric pulse, allowing the uptake of macromolecules that would not otherwise readily enter the cell [51, 52]. Once inside, nuclear access of the RNP is promoted by nuclear localisation signals engineered into the Cas9 protein, enabling rapid, efficient engagement of the host nuclear import machinery and rapid access to the genomic target [52]. This workflow was established using the Neon Electroporation System, but electroporation platforms such as MaxCyte and Lonza 4D-Nucleofector may also be suitable for CRISPR RNP delivery in laboratories with different equipment [53, 54].

**Fig. 8:**
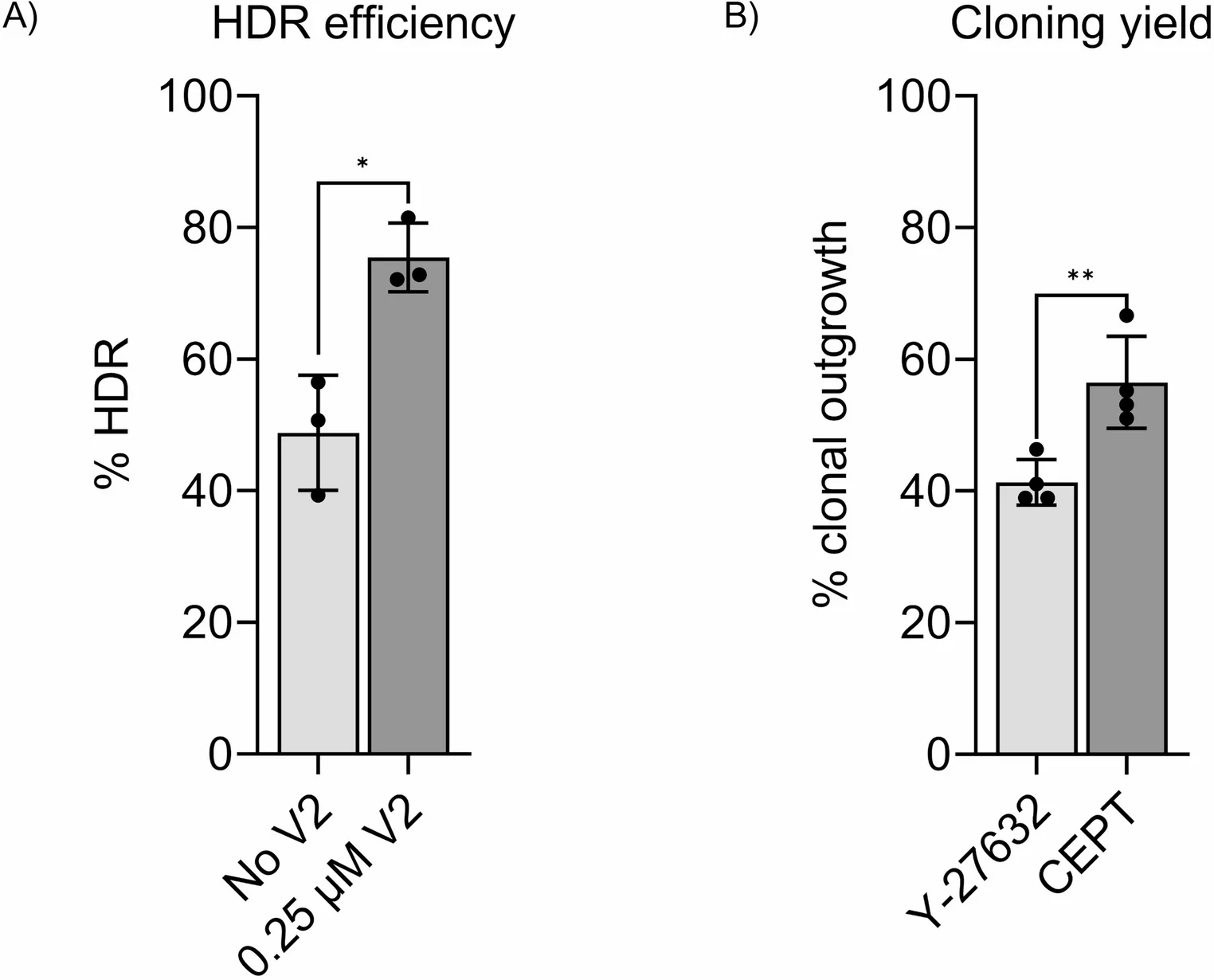
HDR and cloning efficiency. (**A**) Total HDR efficiency in three independent and most recent experiments, in the presence or absence of 0.25 µM HDR Enhancer V2. (**B**) Percentage of clonal outgrowth from the four independent and most recent cloning experiments, using either 10 µM Y-27632 or CEPT supplement. Cloning yield was defined as the number of wells containing single-cell-derived clones relative to the total number of wells seeded. Statistical analyses were performed and graphs were generated with GraphPad Prism 10.2.0. The normal distribution was assessed by Shapiro-Wilk test. For comparison between two groups, statistical significance was determined by unpaired Student’s t-test. A probability value of *p* < 0.05 was considered statistically significant, with * *p* < 0.05 and ** *p* < 0.01

Optimising for high-efficiency homozygous HDR in human iPSCs requires coordinated control of gRNA selection, donor template design, RNP delivery, and post-editing culture. The first critical step is gRNA design, screening and selection. Optimal gRNA selection requires high on-target activity, low off-target potential and close proximity to the edit site. The gRNA scores provided by Benchling and IDT are sequence-based in silico predictors of on-target activity, not direct quantitative measures of experimental editing efficiency. These should be interpreted as tools for relative guide ranking rather than as exact predictors of NHEJ frequency measured by ddPCR. Observed editing efficiency depends on many variables that are not fully captured using in silico prediction algorithms, such as cell type, target-locus architecture, chromatin accessibility, sgRNA biophysical properties, delivery conditions and DNA-repair state. For this reason, we recommend experimental screening of a minimum of five candidate gRNAs per target, in the same target cell type and under the same delivery conditions intended for the subsequent HDR experiments (Fig. 3). Among guides with sufficient activity, generally at least 20% total NHEJ, priority should then be given to those with the highest on-target activity and whose cut site lie closest to the intended edit, because the cut-to-mutation distance strongly affects precise HDR incorporation and is especially critical when aiming for efficient homozygous installation [30, 55, 56]. Specifically, the target mutation should ideally be within 10 bp of the Cas9 cut site to avoid the marked decline in HDR efficiency associated with greater distances [30, 55, 56]. The remaining candidates should then be ranked according to predicted specificity, with preference for guides with a low off-target burden and no high-priority predicted off-target sites in coding or otherwise functionally relevant regions. Thus, gRNA and ssODN design should be considered jointly from the outset, because the chosen gRNA determines which donor design strategy can be used for HDR-mediated gene correction. For example, in the representative experiments reported here for the missense *F7* G240R mutation (c.718G>C, NM_000131.4; hg38 chr13:g.113117509G>C) from Roman et al., 2026 [29], the two most efficient gRNAs were gRNA #2 and gRNA #5, which yielded 41% and 48% total NHEJ, respectively (Fig. 4B). However, gRNA #5 was selected not primarily for its slightly higher on-target activity, but because its cut site was substantially closer to the intended edit, with a cut-to-mutation distance of 6 nt compared with 21 nt for gRNA #2 (Fig. 4). This difference had major implications for donor design. gRNA #5 enabled the use of a negatively oriented 87-nt ssODN with a 2-nt repair window [5′-(…)CT(…)-3′], whereas gRNA #2 required a positively oriented 107-nt ssODN with a 25-nt repair window [5′-(…)G-(25 nt)-C(…)-3′], in which the 3′ edit represents the synonymous PAM-altering variant introduced to prevent re-cleavage (data not shown). When the desired edit does not naturally disrupt the PAM, synonymous blocking mutations should be incorporated into the donor template. This strategy can enhance editing accuracy by up to 10-fold per allele, potentially yielding a 100-fold overall gain in cells where both alleles are corrected [20, 55]. It is particularly important to prioritise designs in which the intended correction itself inherently disrupts the PAM, or otherwise introduces a mismatch within the guide recognition sequence. This is particularly important in heterozygous correction settings, where allele-selective targeting can restrict cleavage to the mutant allele and spare the healthy allele from unnecessary editing. We therefore recommend testing such candidate gRNAs in both the patient-derived mutant line and a healthy control line to assess on-target activity together with discrimination against the wild-type sequence. While synonymous mutations are often termed “silent”, they can significantly impact gene expression and protein function by altering splicing, mRNA stability, translation kinetics, and protein folding [57, 58]. These potential effects underscore the need for careful evaluation when introducing synonymous PAM-altering mutations, for which we have outlined practical guidelines. In addition, donor templates should be tailored in homology arm length, symmetry, and strand orientation for the intended edit. For insertions <120 bp, Alt-R–modified ssODN donors (<200 nt) are preferred over double-stranded DNA (dsDNA) templates, with 30–60 nt homology arms generally providing the highest knock-in efficiency [30, 59]. Strand orientation should be matched to the repair context, with PAM-proximal edits yielding higher HDR when targeting-strand donors are used and PAM-distal edits yielding higher HDR when non-targeting-strand donors are used [30, 59]. When insertions are placed directly at the cut site, no consistent strand bias is typically observed [30, 59]. Alt-R modification of ssODNs can improve stability against exonuclease degradation and increase HDR efficiency compared to unmodified donors, with symmetric homology arms providing the highest performance when such modifications are used [30, 59]. When these design parameters are combined with coordinated optimisation of RNP delivery and post-editing culture conditions, HDR efficiency can be further improved by transiently biasing DNA repair toward the HDR pathway via small-molecule modulators such as HDR Enhancer V2 (Integrated DNA Technologies, IDT) or Enhancer Protein (IDT) [30, 59, 60] (Fig. 8A). Experimentally, we recommend testing multiple conditions in the 0.1-1 µM concentration range in parallel at the initial pool stage, enabling direct comparison of HDR outcome and cell recovery across conditions before selecting the pool to take forward for expansion/clonal isolation. In this study, the optimal working range of Alt-R HDR Enhancer V2 (IDT) balancing editing performance and cell quality was generally 0.1-0.5 µM, while 0.5-1 µM conditions were generally associated with increased cellular stress and apoptosis. Importantly, selection of the HDR-edited pool should prioritise use of the lowest effective enhancer dose, with emphasis on clonal quality and post-editing recovery rather than maximal bulk HDR frequency alone, since excessive cytotoxicity at this stage can compromise the quality of downstream clonal iPSC lines. For example, although 1 µM yielded 50.7% HDR in the representative experiments reported here from Roman et al., 2026 [29], this condition was also associated with marked cell death and cellular stress (data not shown). By contrast, 0.25 µM already increased HDR to 42.3%, compared with 19.4% in the absence of enhancer, while imposing a substantially lower cytotoxic burden on the cells (Fig. 4C). For this reason, we recommend prioritising the 0.1-0.5 µM range, particularly when satisfactory editing efficiencies have already been achieved within this window. Together, these choices can provide practical levers to reduce repair pathway competition, promote simultaneous HDR on both alleles, and enable reproducible recovery of fully edited, homozygous iPSC clones without compromising genomic stability. Lastly, the workflow integrates a stepwise genotyping approach, enabling the precise, quantitative detection of genomic edits. Initial screening of candidate gRNAs is performed using a ddPCR-based NHEJ assay to quantify on-target cleavage efficiency, followed by HDR-specific ddPCR to measure precise knock-in or correction events in bulk-edited populations. This quantitative approach enables absolute measurement of editing outcomes across multiple gRNA-donor designs, culture conditions, and experimental parameters. By screening multiple edited pools, the most effective combination of design/workflow variables can be identified before committing to single-cell cloning. By prioritising these selection criteria, we enhance the probability of obtaining homozygous clones and avoid the unnecessary expenditure of resources on inefficient editing conditions. First, single-cell-derived clones from the selected pool are screened by Sanger sequencing to verify the presence of the intended edit and to exclude unwanted allelic outcomes. Only on confirmation of homozygosity should one proceed to WES, which simultaneously verifies the on-target edit, evaluates overall exome integrity, identifies potential off-target changes within coding regions and provides evidence of monoclonality. Second, complementing conventional G-banding karyotyping with targeted ddPCR/qPCR-based assays and SNP-array/CMA-based copy-number profiling, for example iCS-Digital PSC and iCS-digital Aneuploidy (StemGenomics), and KaryoStat (Thermo Fisher Scientific), respectively, can provide a more comprehensive and robust assessment by combining detection of large chromosomal abnormalities with higher-resolution detection of sub-karyotypic copy-number alterations. Functional assays and differentiation on WES-confirmed clones act as a later stringent functional test, as they can expose latent defects that might otherwise remain hidden, but have the potential to impair or entirely block lineage commitment. Additionally, high-sensitivity genome-wide off-target mapping can be achieved using advanced techniques, such as GUIDE-seq or CIRCLE-seq [32–37]. GUIDE-seq is a sensitive cell-based assay that detects Cas9-induced DSBs through integration of a double-stranded oligodeoxynucleotide (dsODN) tag at cleavage sites, followed by tag-specific amplification and high-throughput sequencing [32–37]. By contrast, CIRCLE-seq is a highly sensitive in vitro method based on CRISPR-Cas9 RNP-mediated cleavage of purified circularised gDNA, without requiring cellular transfection and enabling low-background, genome-wide nomination of candidate off-target sites through sequencing of nuclease-linearised fragments [32–37]. Instead, WGS is used at the final stage of validation, providing a comprehensive overview of genomic integrity, including variants in non-coding regions and larger structural changes, in fully characterised lines. As part of routine quality control, we strongly recommend regular monitoring of genetic stability and karyotypic integrity in all iPSC cultures, particularly in single-cell-derived clonal lines, as recurrent genomic alterations may arise during culture, most commonly as CNVs involving hotspot regions such as 1q, 12p, 17q, 20q, and the X chromosome [61, 62]. By employing this tiered screening approach, we maximise the probability of identifying high-quality and high-performing clones, in a time- and cost-effective manner. Single-cell cloning of iPSCs is technically demanding because dissociation and prolonged handling trigger sensitivity to DNA damage, apoptosis, and unintended differentiation [22, 63]. Conventional approaches, such as manual colony picking or limiting dilution, are labour-intensive and operator-dependent. These methods frequently result in low throughput, compromised cell viability, and insufficient assurance of monoclonality [64–66]. Employment of limiting dilution lacks sufficient statistical confidence in monoclonality at practical plating densities. Furthermore, the high frequency of empty wells significantly increases timelines, operational costs, and the overall handling burden. FACS-based single-cell sorting imposes high shear stress (>2-5 psi) and mechanical damage, often resulting in increased cell damage and low viability [24]. In contrast, integrated microfluidic (Cytena) (Fig. 5) and impedance-based (SEED Biosciences) (Fig. 6) dispensing modalities enable gentle isolation (≤2 psi), real-time single-cell deposition monitoring, higher first-pass success and overall superior results [24]. In particular, UP.SIGHT (Cytena) provides a high level of precision and control over clonal derivation through real-time optical interrogation, enabling cells to be visualised and selected prospectively according to predefined physical parameters such as size and roundness (Fig. 5). This permits highly accurate dispensing, such that only droplets containing a single cell are released into the cloning well, while empty droplets and droplets containing multiple cells are excluded and discarded prior to deposition. Each successful dispensing event is documented by an image series captured at the nozzle and complemented by orthogonal confirmation with the integrated automated imaging system, which enables rapid and efficient full-well imaging across the entire plate (Fig. 5). By contrast, DISPENCELL-S3 (Fig. 6) is inherently dependent on the quality of the input single-cell suspension. The system detects and classifies dispensing events by impedance, using particle and aggregate thresholds to interpret the electrical signal generated as cells pass through the sensing aperture (Fig. 6C). As a result, single-cell deposition efficiency is not constant but may vary substantially between preparations. In our hands, it generally ranged from approximately 50 to 80%, depending on the proportion of true single cells in suspension, which is influenced both by cell-line-specific dissociation behaviour and by technical sample preparation (Fig. 6B). Consequently, multiple cells and closely apposed doublets, triplets or small aggregates may still enter the workflow and need to be identified during downstream review, rather than being prospectively excluded before deposition (Fig. 6B and C). Collectively, these features establish the presented workflow as a practical, scalable alternative to manual picking, limiting dilution, and routine FACS within iPSC genome-editing pipelines. A major advance in the stem cell field was the introduction of Rho-associated coiled-coil forming protein serine/threonine kinase (ROCK) inhibitor, Y-27632 [67]. In their landmark 2007 study, Watanabe et al. [67] demonstrated for the first time that transient treatment with ROCK inhibitor Y-27632 markedly reduced dissociation-induced apoptosis in human embryonic stem cells (hESCs). However, prolonged exposure has also been reported to be associated with metabolomic alterations and, potentially, with variability in iPSC differentiation potential [68, 69]. Nevertheless, poor survival and cell quality after single-cell dissociation and cloning remained a major roadblock for iPSC genome editing until recently, indicating that the complexity of dissociation-associated stress extends beyond the ROCK pathway alone. 

In our initial experiments, cloning was performed using complete E8 medium in combination with Y-27632, as iPSCs were routinely maintained in E8 medium. However, this required daily medium replacement and consistently coincided with low survival and poor clonal recovery. Recognising the susceptibility of seeded iPSCs to early-stage stress, we conducted a systematic reassessment of cloning variables to improve survival and expansion rates. Furthermore, pilot experiments used iPSC lines that were cultured on Geltrex or vitronectin (VTN-N) extracellular matrix. Geltrex proved suboptimal for high-throughput single-cell cloning, also possibly due to challenges in reproducible coating and limited stability during 96-well plate preparation. In addition, it is not xeno-free and therefore not compatible with translational cell-therapy manufacturing pipelines, supporting the use of defined recombinant matrices such as vitronectin instead. We addressed these vulnerabilities by minimising mechanical/environmental disturbances during the initial 24-hour recovery window, the most critical phase following single-cell isolation and seeding. To minimise early-stage disturbance, we employed StemFlex medium, which supported a 48-hour recovery window without additional feeding, due to its stabilised FGF-2, which ensured maintenance of pluripotency and undifferentiation during this critical phase. Notably, the cells were directly transitioned into StemFlex at the time of dispensing without prior adaptation to simplify the experimental workflow, with no adverse effects on survival or clonal outgrowth. Additionally, we replaced Y-27632 with CultureCEPT Supplement (Thermo Fisher Scientific) (Fig. 8B), which has been reported to provide comprehensive cytoprotection against the major forms of cellular stress and DNA damage, while improving survival and clonal expansion [24, 31]. CultureCEPT Supplement (Thermo Fisher Scientific) is an innovative cytoprotective formulation based on the small-molecule cocktail developed by Chen et al., 2021 [31], comprising Chroman 1, Emricasan, Polyamines and Trans-ISRIB (CEPT). Chroman 1 is a highly potent and selective ROCK2 (Rho-kinase) inhibitor that functionally outperforms conventional Y-27632, thereby counteracting dissociation-induced actomyosin hyperactivation and excessive cell contraction [24, 31]. Emricasan, a pan-caspase inhibitor, complements ROCK inhibition by suppressing caspase-dependent apoptosis and acts synergistically with Chroman 1 to further improve cell survival [24, 31]. The polyamine component enhances the viability and resilience of iPSCs by supporting cell growth and stabilising cellular structures and macromolecules [24, 31]. Trans-ISRIB, a potent inhibitor of the integrated stress response (ISR), further broadens cytoprotection by attenuating ISR signalling and helping preserve protein translation under acute culture stress [24, 31]. 

### Limitations

Although this workflow was validated on multiple iPSC lines (1 healthy donor and 7 patient-derived iPSCs), genes (*F7*, *SERPINC1* and *UNC45A*), and mutations, performance should be expected to vary with genetic background, locus context, chromatin accessibility, and specific guide activity [18, 38, 39]. Also, automated single-cell dispensers may not be standard equipment in many laboratories, as the instruments can be expensive and other methods such as manual colony picking, limiting dilution, and FACS isolation are comparatively low-cost and broadly accessible. However, if any of these other methods is well established in a laboratory, it might be implemented in this protocol, although the yield may differ for the aforementioned reasons [22, 24, 25, 70, 71]. This protocol was optimised under defined xeno-free conditions. Matrix or medium substitutions, growth factor and reagent stability, and plate layout can affect clonogenicity and edit outcomes, and these variables were not exhaustively benchmarked. This study focuses on small, precise HDR edits. Large knock-ins with long dsDNA donors, safe-harbour integrations, and complex allelic rewrites likely require additional template engineering, delivery strategies, or selection steps that were not evaluated. Several interventions that bias repair toward HDR have been reported in previous studies [17, 18, 20, 21, 28, 30, 50, 52, 55, 56, 59, 72].

Two significant and initially unexpected events related to CRISPR-mediated gene correction were identified during the development and optimisation of this workflow, both highlighting important pitfalls in assay design and screening strategy. First, when using donor templates containing two separate nucleotide mismatches, i.e., both a PAM-altering variation (4-5 nucleotides (nt) proximal) and a distal (21-29 nt) disease-correcting point mutation, we observed a marked imbalance in incorporation frequency between the two edits. For example, with the PAM-altering variant positioned at 5 nt from the cut site and the desired edit at 29 nt, we obtained ~12.5% edited clones, whereas the remaining fraction was completely unedited (data not shown). Within this ~12.5% positive fraction, ~80% carried biallelic incorporation of the PAM-altering variation only, while the remaining ~20% harboured both the PAM-altering variant and the desired edit (data not shown). In one instance where the desired edit was located slightly closer to the cut site (21 nt cut-to-mutation distance), we detected ~17.5% of clones with biallelic integration of the PAM-altering variation but only monoallelic incorporation of the desired edit, indicating complete editing at one allele and partial editing of the other, favouring the PAM-altering variant (data not shown). These partial integration events were surprising but consistent with previous observations that HDR efficiency decreases sharply with increasing distance from the DSB, favouring incorporation of proximal donor mismatches [73, 74]. Importantly, this effect was initially obscured by a biased assay readout, as the HDR ddPCR assay was automatically designed by the software to only recognise the PAM-altering mutation. This assay design limitation was present at the time these experiments were performed, but has since been addressed through improvement of the Bio-Rad ddPCR HDR assay design tool, which now allows undesired nucleotides to be masked using “N”. This provides a practical means of circumventing the issue by excluding unwanted edits from detection and enabling selective interrogation of one of two distal edits when only one is intended to be measured. As a result, donor integration events lacking the disease-correcting mutation were falsely scored as successful HDR. This discrepancy became evident upon Sanger sequencing, which clearly resolved the true allele-level frequencies of both mismatches and revealed frequent partial donor incorporation (Supplementary fig. 2A). Based on this observation, we recommend that when donor templates introduce multiple mismatches at different distances from the Cas9 cut site, HDR assay design should explicitly target the intended disease-correcting mutation, rather than defaulting to detection of a proximal PAM-altering change. Verification of PAM disruption should then be performed as a secondary validation step during downstream clone characterisation. 

Second, we observed rare instances of single-cell-derived clones harbouring large (~485 nt) deletions following CRISPR-Cas9 editing (Supplementary fig. 2B). These events were not detected during initial screening by PCR-based Sanger sequencing, as one of the primers annealed within the region that was later found to be deleted, causing preferential monoallelic amplification of the intact allele. Consequently, Sanger sequencing yielded a false-positive result by interrogating a single allele and masking the presence of the deletion (Supplementary fig. 2B). The discrepancy became apparent only upon downstream WES analysis (Supplementary fig. 2C). However, the detection required careful manual inspection of the alignment data, as reads spanning the deletion were automatically soft-clipped during alignment and therefore not readily visible unless the option to display soft-clipped bases was explicitly enabled in the software. More broadly, unintended events may extend beyond the monoallelic deletions observed here and include larger segmental deletions, complex rearrangements and loss of heterozygosity (LOH) [75, 76]. These findings were consistent with previous evidence by Simkin et al., 2022 [77], who showed that apparently homozygous CRISPR/Cas9-edited iPSC clones may in fact be hemizygous because of large monoallelic on-target alterations. Interestingly, Simkin and colleagues examined 27 iPSC clones generated after targeting 9 loci and found that about 33% carried unintended defects that escaped standard PCR and Sanger sequencing analysis [77]. As a result, quantitative genotyping PCR (qgPCR) assays were used to evaluate the targeted allele copy number as an initial screening approach [77]. To reduce the risk of advancing single-cell-derived clones harbouring large unintended genomic alterations into downstream analyses, we therefore recommend accounting for this possibility initially when designing PCR-based screening strategies, for example by targeting larger genomic regions spanning the edit site or by designing two partially overlapping amplicons, to minimise allele-specific amplification bias. Importantly, we strongly recommend that qgPCR-based copy number assays and broader sequencing-based approaches, such as WES, are always systematically incorporated as part of clone validation, as they provide an essential safeguard against rare but consequential genomic alterations that may evade conventional PCR-dependent screening. In the present study, WES was used as an intermediate clonal-validation step, but it should not be regarded as a comprehensive off-target discovery assay. WES is limited to exonic regions and, unlike final WGS, it does not enable broader assessment of non-coding variants or larger structural alterations across the genome. Instead, a more comprehensive evaluation of genomic integrity can be achieved by combining G-banding with ddPCR/qPCR-based and SNP-array/CMA-based copy-number analyses. In addition, off-target assessment beyond the exonic space can be strengthened by complementary unbiased and sensitive methods, such as CIRCLE-seq [32–37]. 

This workflow integrates several optimised parameters to facilitate precise HDR-based editing, including refined CRISPR oligonucleotide design, electroporation of HiFi Cas9 RNP complexes, and the use of HDR enhancers. However, the study was not designed to benchmark these interventions against alternative chemistries or dosages, and the individual contribution of each component remains undetermined from the current data. Also, the effects of such interventions may vary by locus and iPSC line and may influence cell physiology and the distribution of editing outcomes. These context-dependent effects were not evaluated systematically in this study.

## Conclusion

In summary, this method replaces slow, operator-dependent clonal isolation with a comprehensive, efficient and scalable pipeline for generating, screening, and expanding high-quality monoclonal iPSC lines following CRISPR-Cas9 genome editing, thereby streamlining the development of disease models and therapeutic platforms across a wide range of basic and translational applications.

## Supplementary Information

Below is the link to the electronic supplementary material.

Supplementary Material 1 (PDF 565 KB)

Supplementary Material 2 (XLSX 14.3 KB)

## Data Availability

The data analysed in this study are available from the corresponding author upon reasonable request.

## Abbreviations

Rxn: reaction
RT: room temperature
