# Supplementary material for: From Design to Single-Cell Cloning: A Complete RNP-Based CRISPR-Cas9 Protocol for Precision Gene Correction in Human iPSCs
Source: Stem Cell Rev Rep. 2026 Aug 31;22(8):3790–816. doi: 10.1007/s12015-026-11221-6 (PMC13719729; doi:10.1007/s12015-026-11221-6)
Supplement: Supplementary file 1 [file 12015_2026_11221_MOESM1_ESM.pdf]

Supplementary information

From design to clonal isolation: an RNP-based workflow for precise CRISPR-Cas9–mediated gene correction in human iPSCs

This document includes:

- Supplementary figures and figure legends
- Troubleshooting

Supplementary figures

Supplementary figure 1

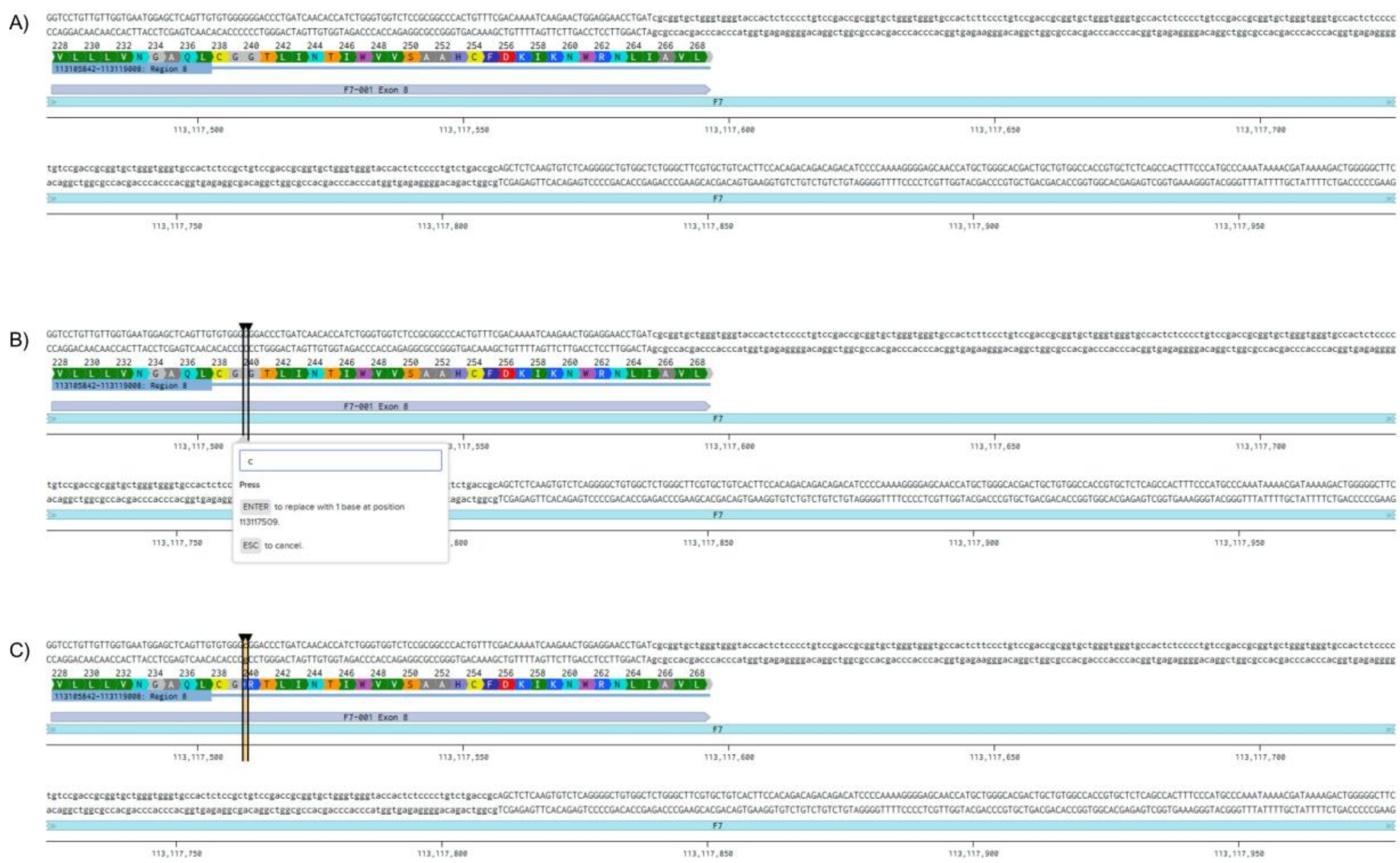

Supplementary figure 1. Sequence maps of the wild-type and mutant *F7* gene sequences. (A) Import and visualization of the wild-type *F7* gene sequence on Benchling. (B) Introduction of the patient mutation, i.e., missense variant c.718G>C (NM_000131.4) (GRCh38 chr13: g.113117509G>C, p.G240R) in the *F7* gene. (C) Confirmation of the mutated *F7* sequence map and the alteration of the amino acid sequence (p.G240R).

Supplementary figure 2

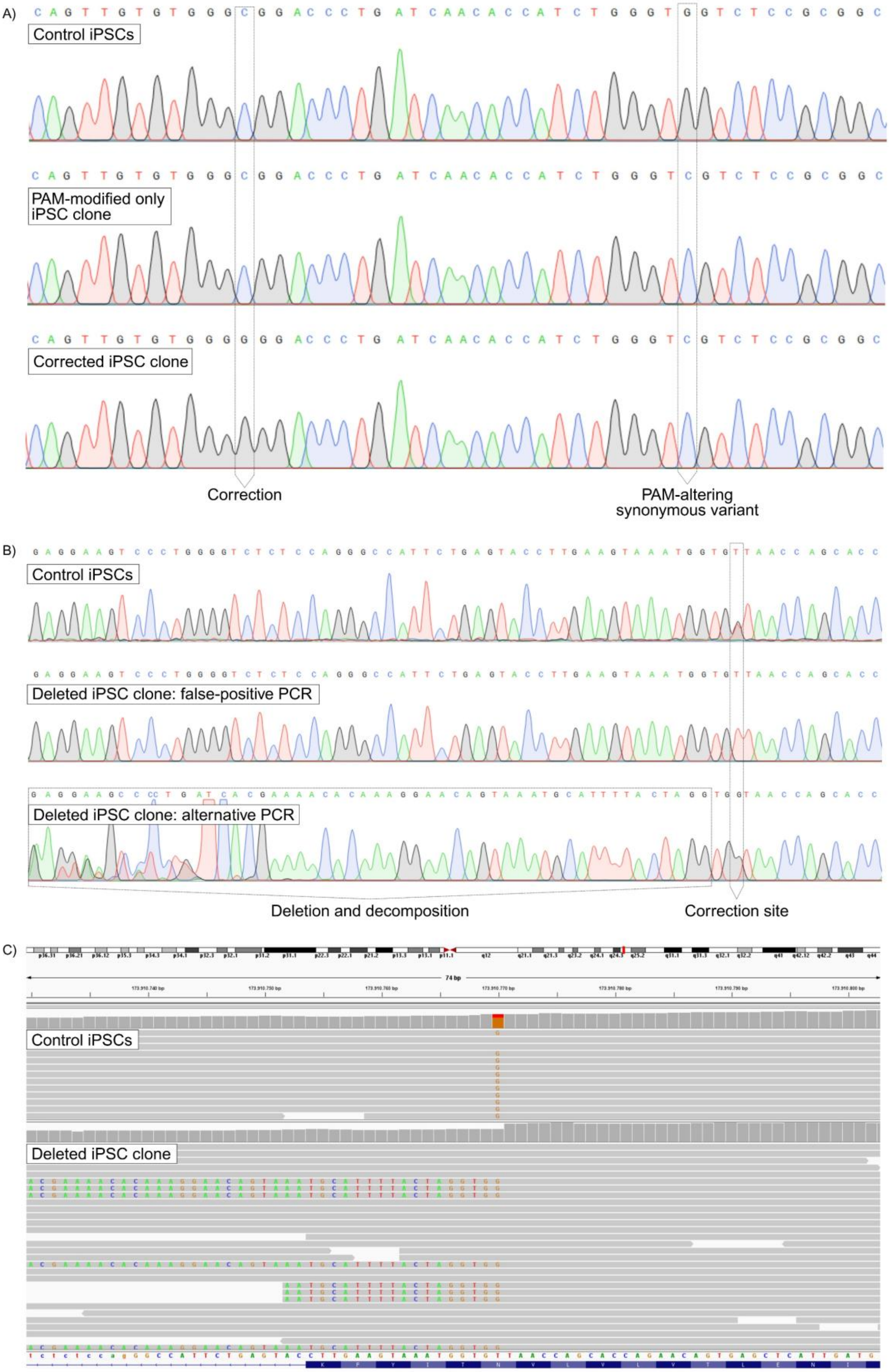

Supplementary figure 2. Imbalanced incorporation of parallel edits and rare monoallelic deletions highlight pitfalls in CRISPR-mediated assay design and screening strategies. (A) Sanger sequencing view of a 25-nt-wide repair locus in exon 8 of *F7*. The panels show the unedited iPSC control (top), a single-cell-derived clone harbouring only the PAM-altering synonymous variant (middle) and a homozygously corrected iPSC clone carrying both the PAM alteration and the disease-correcting mutation (bottom). (B) Sanger sequencing of a single-cell-derived iPSC clone carrying an ~485-nt deletion at the CRISPR-targeted locus (*SERPINC1*, exon 4). The panels show the unedited iPSC control harbouring a heterozygous missense variant (T>G) (top), a false-positive Sanger result arising from preferential monoallelic PCR amplification (middle), and sequencing result obtained using a wider PCR target region (bottom), which reveals the aberrant sequence and the decomposition window indicative of the large deletion. (C) Integrative Genomics Viewer (IGV; igv.org) view of data from the Illumina Clinical Research Exome - CRE V4 analysis. Sequencing reads are presented in grey, with the GRCh38/hg38 reference sequence displayed at the bottom. The panels show the unedited iPSC control (top) and the iPSC clone with the monoallelic deletion (bottom).

Troubleshooting

Workflow stage	Observation	Possible causes	Recommended solutions
**Design of the CRISPR-Cas9 editing components**	Not possible to introduce a PAM mutation in the selected ssODN donor template	Sequence constraints prevent a synonymous change; PAM modification fails the validation process	Introduce the repair track mutations suggested by the IDT HDR Design Tool or manually design repair track changes in Benchling HR design tool; consider selecting alternative gRNAs with similar predicted efficiency that allow for a PAM mutation; if neither option is feasible, proceed without PAM alteration
	Not possible to design a gRNA in close proximity to the desired edit site	Sequence context limits available NGG PAMs near the correction site; the selected gRNA cut site too distant for efficient biallelic HDR	Ensure that the HDR ddPCR detection assay targets the precise correction site rather than the PAM sequence; prepare to screen a larger batch of single-cell-derived iPSC clones
**Thawing iPSCs**	Low viability after thawing	Osmotic shock during dropwise medium addition; exposure to air; overconfluence pre-freeze; improper freezing technique	Add complete medium supplemented with CultureCEPT slowly while gently rocking the tube; keep the pellet covered with medium at all times; freeze iPSCs at 60-75% confluence; refine the freezing procedure (e.g., EDTA incubation)
	Uneven attachment or detachment within 18 h	Lack of proper cell distribution across the well surface prior to incubation; movement of the plate during initial incubation; cell stress during thawing (e.g., osmotic shock,	Distribute cells evenly by gently rocking the plate side-to-side and back-and-forth immediately after seeding; keep the plate stationary for the full 18 h attachment period and avoid repeated cabinet door

		prolonged exposure to DMSO); cell stress during freezing (e.g., improper freezing technique)	openings; minimize handling stress during thawing; ensure controlled-rate freezing and thorough mixing of cryoprotectant before freezing
**Expansion and culturing of iPSCs**	Reduced growth after passaging	Overexposure to EDTA; mechanical damage during transfer; suboptimal VTN coating quality (e.g., expired, uneven, dried before use); inappropriate splitting ratio; use of high-passage or stressed cells; suboptimal confluence and/or colony size at the time of passaging	Empirically optimize EDTA incubation (usually 2-4 min); ensure VTN coating is fresh, uniform, and kept hydrated until use; use an appropriate split ratio (e.g., 1:4 for healthy cultures in the exponential growth phase); work with low-passage iPSCs for optimal growth and genetic integrity; gently dispense medium along the side wall to minimize shear stress
	Insufficient aggregate size and suboptimal attachment after 24 h	Overexposure to EDTA; mechanical stress during transfer; use of high-passage or stressed cells; suboptimal confluence and/or colony size at the time of passaging	Empirically optimize EDTA incubation (see previous); aim for 50–200 μm aggregates and transfer suspensions gently to avoid shear stress; work with healthy, low-passage iPSCs at 60-75% confluence, when colonies are uniformly sized
**Freezing iPSCs**	Suboptimal detachment when freezing multiple wells in the same round	Prolonged or staggered EDTA exposure due to sequential handling; repeated incubator entry/exit during EDTA incubation causing temperature fluctuations; increased idle time between aspiration and resuspension	Avoid freezing too many wells in a single round and limit each round to 3-4 wells to minimize prolonged EDTA exposure; when planning both freezing and passaging, designate separate wells for each purpose (e.g., dedicate 1-2 wells for passaging and the rest for freezing); minimize the number of times plates are moved in/out of the incubator during

			EDTA treatment; work swiftly but gently to prevent drying of the well surface before resuspension
**Gene editing of iPSCs**	Poor or no editing	Suboptimal gRNA design or distance from the intended edit site; degraded/incorrectly stored CRISPR components; suboptimal RNP complexing; cell quality (viability, confluence) below threshold; incorrect Neon settings	Re-verify gRNA sequence, strand/orientation and cut-site proximity to the intended edit; design and test an alternative gRNA closer to the edit; use fresh, correctly stored reagents and limit freeze-thaw cycles; anneal crRNA:tracrRNA correctly (or use pre-assembled sgRNAs (an approach not used in this study)) and form the RNPs immediately before use (30 minutes); use iPSCs at 60-75% confluence and ensure that cell viability at count is ≥90%; ensure correct Neon settings
	Low cell recovery	Prolonged Accutase exposure; mechanical stress/damage during resuspension; suboptimal post-nucleofection density; excessive time in Resuspension Buffer R; excessive Enhancer V2 concentration; gRNA-specific toxicity	Optimize Accutase exposure time; resuspend cells gently; ensure correct counting of viable cells; increase cell density post-nucleofection by reducing well surface area; reduce time in Resuspension Buffer R to the minimum; optimize Enhancer V2 dose; use an alternative gRNA with similar on-target performance
	Inconsistent outcomes and/or technical failures	Air bubbles or underfilled Neon tip; inaccurate cell count; CRISPR reagent degradation; reaction cross-contamination;	Inspect for bubbles before pulsing and ensure the tip is filled to the correct volume and seated firmly in the holder; ensure correct counting of viable cells;

		mycoplasma contamination	use fresh, properly stored CRISPR reagents (see previous); use fresh Electrolytic Buffer and avoid reusing the same Neon Tube when running different, unrelated reactions or cell lines; confirm mycoplasma-negative status before editing
**NHEJ/HDR detection by ddPCR**	Assay not designable	Sequence constraints or complexity prevent probe/primer placement	Use the T7EI assay or alternative validated screening methods for on-target cutting activity detection (NHEJ) or use Sanger sequencing, WES or alternative validated screening methods for detection of donor template insertion (HDR)
	Suboptimal or failed target amplification	Suboptimal annealing/extension time; template quality issues; incorrect restriction digestion approach or enzyme; presence of inhibitors and/or contaminants	Increase the annealing/extension time to 6 minutes and further optimize by gradually reducing the annealing/extension time in 1-minute intervals to identify the minimal optimal annealing/extension time; verify template integrity before use; when restriction digestion is required, use one of the recommended enzymes according to guidelines; change restriction enzyme; avoid conditions that could introduce inhibitors
	Suboptimal HDR event detection	Probe targets the PAM mutation instead of the intended knock-in site, which lies far from the cut site	Design the assay to target the intended knock-in or correction site rather than the PAM sequence (PAM mutations are incorporated more frequently due to their proximity to the cut site)

**Single-cell cloning of iPSCs**	Low clonal yield and recovery	Suboptimal cell quality and viability; prolonged Accutase exposure; mechanical stress/damage during resuspension; extended time outside the incubator or prolonged dispensing	Use healthy iPSCs that have fully recovered from nucleofection, in the exponential growth phase, at 60-75% confluence, and with viability ≥90%; optimize Accutase exposure time; resuspend cells gently; ensure correct counting of viable cells; use the recommended cell density during dispensing to avoid prolonged dispensing and extended time outside the incubator; avoid plate movement and disturbance for ≥48 h
	Uneven clone growth and/or morphology	Stress during dispensing; suboptimal cell quality and viability; biological effects of genome editing (e.g., gRNA-specific effects, target gene function); mycoplasma contamination	Minimize mechanical and thermal stress during dispensing and ensure iPSCs meet quality criteria (see previous); review and, if necessary, modify editing conditions or select an alternative gRNA; ensure colonies remain covered with medium at all times to prevent drying or sudden temperature stress; perform regular mycoplasma testing
**Screening and expansion of the clonal iPSCs**	Poor recovery after initial expansion	Clones expanded too soon after single-cell seeding; residual stress from cloning; lack of CEPT or ROCK inhibitor	Allow sufficient recovery time before the first expansion; use CEPT or 10 μM Y-27632 supplementation for 18 h during the first expansion into a 24-well plate
